# *In silico* coronary wave intensity analysis: application of an integrated one-dimensional and poromechanical model of cardiac perfusion

**DOI:** 10.1007/s10237-016-0782-5

**Published:** 2016-03-23

**Authors:** Jack Lee, David Nordsletten, Andrew Cookson, Simone Rivolo, Nicolas Smith

**Affiliations:** Department of Biomedical Engineering, King’s College London, 3rd Floor, Lambeth Wing, St Thomas’ Hospital, London, UK

**Keywords:** Wave intensity analysis, Cardiac perfusion, Poromechanics, Computational modelling

## Abstract

Coronary wave intensity analysis (cWIA) is a diagnostic technique based on invasive measurement of coronary pressure and velocity waveforms. The theory of WIA allows the forward- and backward-propagating coronary waves to be separated and attributed to their origin and timing, thus serving as a sensitive and specific cardiac functional indicator. In recent years, an increasing number of clinical studies have begun to establish associations between changes in specific waves and various diseases of myocardium and perfusion. These studies are, however, currently confined to a trial-and-error approach and are subject to technological limitations which may confound accurate interpretations. In this work, we have developed a biophysically based cardiac perfusion model which incorporates full ventricular–aortic–coronary coupling. This was achieved by integrating our previous work on one-dimensional modelling of vascular flow and poroelastic perfusion within an active myocardial mechanics framework. Extensive parameterisation was performed, yielding a close agreement with physiological levels of global coronary and myocardial function as well as experimentally observed cumulative wave intensity magnitudes. Results indicate a strong dependence of the backward suction wave on QRS duration and vascular resistance, the forward pushing wave on the rate of myocyte tension development, and the late forward pushing wave on the aortic valve dynamics. These findings are not only consistent with experimental observations, but offer a greater specificity to the wave-originating mechanisms, thus demonstrating the value of the integrated model as a tool for clinical investigation.

## Introduction

Impaired myocardial blood flow underlies a wide range of cardiac diseases. Notably, there are $\sim$3M coronary angiographies undertaken every year to diagnose coronary artery disease in Europe alone (Cook 2011). The ischaemic cascade, which begins with a perfusion deficit and progresses through mechanical and electrical dysfunction to ultimately culminate in myocardial infarction, has been well documented (Nesto and Kowalchuk 1987). In addition, myocardial ischaemia has received attention as a secondary contributor to several other cardiac diseases including hypertrophic cardiomyopathy (Maron et al. 2009), aortic stenosis (Rajappan et al. 2003) and heart failure (Nakanishi et al. 2012). Perfusion deficits can be brought on by disparate causes, including stenotic lesions in large vessels or microvascular dysfunction, as well as the increase in extravascular compression which is known to heighten subendocardial vulnerability to ischaemia (Hittinger et al. 1995; Heusch 2008). Due to this physiological complexity, the differential diagnosis and the therapeutic trajectories must be pieced together from additional clinical evidences. Accordingly, there has been much interest in a diagnostic approach which would unite the currently overlooked assessments of coronary microcirculation and perfusion–contraction crosstalk into the existing routine clinical workflow.

One technique which has demonstrated potential as an integrated measure is wave intensity analysis (WIA) (Parker and Jones 1990; Parker 2009). The theoretical development of this approach was based on a linearised one-dimensional approximation of wave propagation in elastic arteries, and the characteristic analysis of the resulting hyperbolic system. From simultaneous pressure and velocity waveforms measured via coronary catheterisation, this technique enables the assessment of the combined myocardial and haemodynamic functions within a single diagnosis (Davies et al. 2011; Kyriacou et al. 2012). Recently, a landmark predictive application of WIA was demonstrated for the first time in de Silva et al. (2013), which showed that a real-time WIA-derived index can be used to predict the functional recovery following myocardial infarction. Nonetheless, this study, as well as all other coronary WIA investigations, is currently encumbered by the lack of a method with which to relate WIA observations to the mechanistic foundations underlying the pathophysiology. Without the ability to attribute the altered coronary flow and myocardial dynamics to the chain of events that generate the coronary waveforms, the reverse process of mapping these signals to a specific underlying disease process remains in the domain of qualitative trial and error.

As an alternative to this approach, the convergence of medical imaging with computational modelling allows subject-specific coronary WIA to be studied from a causal mechanistic basis. This is demonstrated in the current work using an animal subject. We require such a model to consist of coronary flow and myocardial mechanistic components coupled together, as the coronary waves are produced by their interaction. To tractably model the coronary circulation necessitates a multiscale strategy, whereby the inertia-dominated flow in large conducting vessels and the predominantly viscous flow in the distal circulation employ alternative modelling paradigms (Lee and Smith 2012). The one-dimensional vascular flow modelling framework has been well established in the literature in the past decade and has been shown to reproduce wave propagation behaviour with high accuracy (Vosse and Stergiopulos 2011). In this work, we combine and extend our previous studies in coupled one-dimensional flow–myocardium (Smith et al. 2002) and poroelastic perfusion modelling (Cookson et al. 2012).

The theory of poromechanics provides an apt framework for perfusion modelling, as it forgoes the need for explicit microvascular geometries and innately addresses crosstalk effects with spatial variability. In the standard poroelasticity, the macro-scale elasticity and fluid conservation laws are derived based on general assumptions without knowledge of the detailed microstructure (Bowen 1980). This is in contrast to the alternative approach of mathematical homogenisation which rigorously determines the governing macroscopic laws and effective constants directly from a specific micro-scale geometry (Cioranescu and Donato 1999). However, although several studies have employed homogenisation to vascularised tissues (Chapman et al. 2008; Shipley and Chapman 2010; Rohan and Cimrman 2010) including those in the large-deformation regime (Rohan 2006; Rohan and Lukes 2011), the requirements of microstructural periodicity and the necessity of numerical solution of the cell problem at every Gauss point currently limit the utility of this approach in large domains (Rohan and Lukes 2011). Accordingly, studies aiming to investigate the perfusion dynamics of the whole heart (Rossi 2007; Chapelle et al. 2010; Cookson et al. 2012) have applied large deformation poroelasticity Biot (1972); Coussy 1995, 2004); Boer 2005). It should be mentioned also that although the theory deals exclusively with macroscopic quantities, the micro–macro averaging technique (Whitaker 1986) has retrospectively provided theoretical rigour to the fundamental governing laws and remains an indispensable tool for characterising the macroscopic properties of a specific physical medium with known microstructure.

It is noteworthy that, to date, the most advanced application of the porous perfusion models has yet to achieve a clinically relevant milestone. Key remaining challenges include the development and validation of a suitable cardiac poroelastic constitutive law, characterisation of hydraulic permeability tensors through coronary microvascular analysis, and an effective strategy for posing boundary conditions on the porous flow domain. While the former two tasks represent an ongoing programme of research beyond the current scope, in this article, we will approach the boundary condition issue in a data-driven manner, i.e. through explicit image-based modelling of the flow in proximal vessels, thus capturing the complex spatial distributions of the feeding sites. Consequently, the presence of the major coronary vessels in the model allows WIA to be performed *in silico*, which will in turn benefit from the increased realism of the distal flow conditions and perfusion–contraction coupling.

The principal objective of this work can thus be summarised as the establishment of an integrated multiscale computational model of myocardial blood flow that incorporates the effects of perfusion–contraction crosstalk. Its utility as a physiological research platform is illustrated through an *in silico* application of wave intensity analysis under various perturbed conditions, thus allowing the direct wave dependence on individual myocardial and haemodynamic parameters to be established.

## Materials and methods

**Fig. 1:**
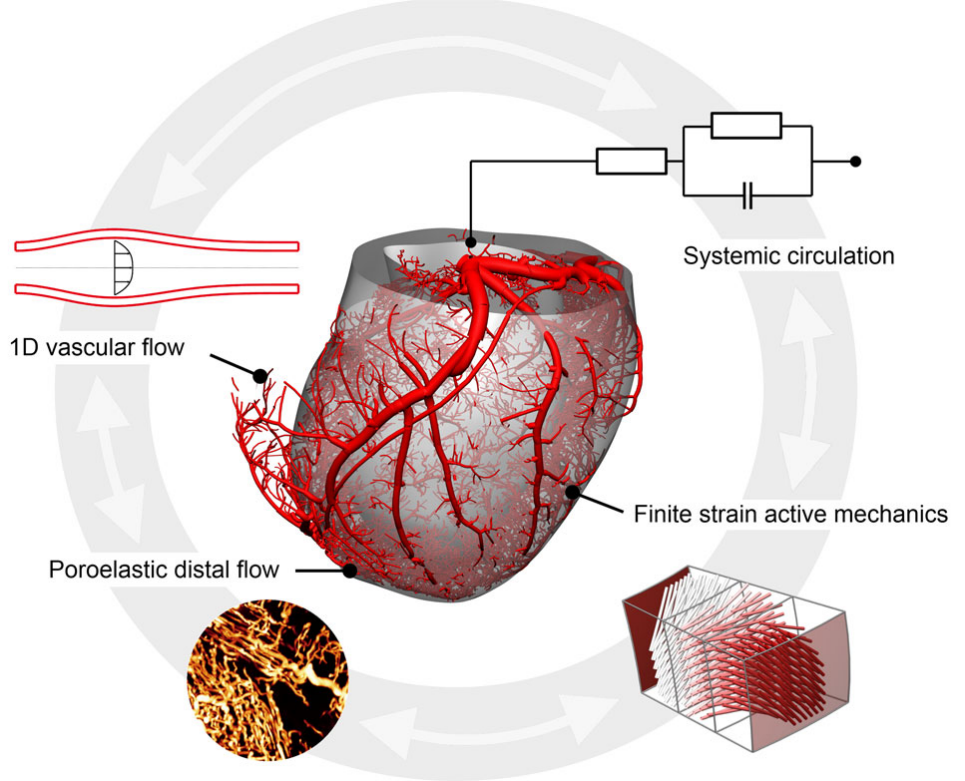
A schematic overview of the integrated perfusion model

The integrated computational model described herein consists of four major components (see Fig. 1), encompassing passive and active myocardial mechanics, coronary flow and myocardial perfusion.

### Poromechanics

*Porous material* In this work, cardiac tissue is idealised as a saturated porous medium consisting of a solid phase (myocardial tissue) and a fluid phase (blood). To proceed, the microstructural particularities are disregarded, and the two constituents are conceptualised to exist co-spatially with varying volume fractions, while retaining their individual properties. Thus, in the following discussions, we refer to the macroscopic solid continuum (referred to as the ‘skeleton’) and the fluid continuum. This concept had been firmly consolidated by the time the classical work of Biot (1941) and mixture theory Truesdell (1957); Truesdell and Noll 1965) arrived, and allows us to consider a solely macroscopic framework.

Denoting a representative elementary volume (REV) as $\text {d}\varOmega$, we can define the respective initial and current Eulerian porosities as the fluid volume fraction, i.e.

1 $$\begin{aligned} \phi _o = \frac{\text {d}\varOmega ^f_o}{\text {d}\varOmega _o}, \quad \phi = \frac{\text {d}\varOmega ^f}{\text {d}\varOmega } \end{aligned}$$

where the subscript *o* and superscript *f* correspond to reference configuration and fluid, respectively. For convenience, we also denote the solid fraction as $\phi ^s \,\,(=1-\phi )$. Note that in describing the deformation of the total porous medium, it is convenient to exploit the skeleton deformation since it is directly observable (Coussy 1995). Therefore, the term *Lagrangian* implies *with respect to the skeleton*.^1^ Following the standard definitions of large deformation kinematics, the material initially located at **X** in the reference configuration is identified with the deformed position **x**, uniquely mapped by

2 $$\begin{aligned} \mathbf{x} = \chi (\mathbf{X},t) = \mathbf{X} + \mathbf{U}(\mathbf{X},t) \end{aligned}$$

where **U** denotes the displacement vector. The deformation gradient tensor ${\mathsf {F}}$ is defined as

3 $$\begin{aligned} {\mathsf {F}} = \frac{\partial \mathbf{x}}{\partial \mathbf{X}} = \nabla _{\mathbf{X}}{} \mathbf{U} +{\mathsf {I}} \end{aligned}$$

leading to the right Cauchy–Green strain tensor ${{\mathsf {C}}}={\mathsf {F^{T}F}}$ and Jacobian $J=det({\mathsf {F}})$. The fluid flow through the porous medium can thus be described in terms of the Darcy velocity $\mathbf {w}$,

4 $$\begin{aligned} \mathbf {w} = \phi \left( \mathbf {V}^{\mathbf {f}}-\mathbf {V} \right) \end{aligned}$$

which is the velocity of fluid $(\mathbf {V}^{\mathbf{f}})$ relative with respect to that of the skeleton $(\mathbf {V}=\frac{\text {d}\mathbf {x}}{\text {d}t})$ weighted by the porosity. Darcy velocity can be expressed in the Lagrangian frame by introducing the following identity relating the flow through an infinitesimal oriented area element $(\mathbf {n}\mathrm{d}a)$

5 $$\begin{aligned} \mathbf {W}\cdot \mathbf {N}\text {d}A = \mathbf {w}\cdot \mathbf {n}\text {d}a \end{aligned}$$

which, in combination with Nanson’s formula, gives

6 $$\begin{aligned} \mathbf {W}(\mathbf {X},t) = J{\mathsf {F^{-1}}}\mathbf {w}. \end{aligned}$$

*Particle and material derivatives* To examine the conservation of physical quantities, two types of derivatives particular to the porous medium can be defined. We focus on the Lagrangian formulation in the following. A *particle derivative* with respect to a solid or a fluid particle is the time derivative that an observer attached to the particle would derive (Coussy 2004). Let $g(\mathbf {x},t)$ be the Eulerian volume density of a physical quantity in the current configuration. We can write

7 $$\begin{aligned} g(\mathbf {x},t) \mathrm{d}\varOmega = G(\mathbf {X},t) \mathrm{d}\varOmega _o \end{aligned}$$

for the corresponding Lagrangian particle density $G({\mathbf {X}},t)$. Now denoting the volume integral ${\mathscr {G}}$ by

8 $$\begin{aligned} \mathscr {G} = \int _\varOmega g(\mathbf {x},t) \mathrm{d}\varOmega \end{aligned}$$

it follows from Eq. (7), the particle derivative is expressed

9 $$\begin{aligned} \frac{\text {d}{\mathscr {G}}}{\text {d}t} = \int _{\varOmega _o} \frac{\text {d}G}{\text {d}t} \text {d}\varOmega _o. \end{aligned}$$

The material derivative $\frac{\text {D}}{\text {D}t}$ accounts for the incoming flux of the considered quantity carried by the fluid, additionally to the solid particle derivative. Hence,

10 $$\begin{aligned} \frac{\text {D}\mathscr {G}}{\text {D}t} = \frac{\text {d}\mathscr {G}}{\text {d}t} + \int _{\partial \varOmega _o} G^{f}\mathbf {W}\cdot {\mathbf {N}} \text {d}A \end{aligned}$$

where $G^{f}(\mathbf {X},t)$ denotes the Lagrangian density function of the fluid particle, which is located by the *skeleton* position vector $\mathbf {x}=\mathbf {x}(\mathbf {X},t)$. By applying (9) and divergence formula to the above, we obtain

11 $$\begin{aligned} \frac{\text {D}\mathscr {G}}{\text {D}t} = \int _{\varOmega _o} \left[ \frac{\partial G}{\partial t}+\nabla _{\mathbf {X}} \cdot (G^{f}\mathbf {W}) \right] \mathrm{d}\varOmega _o. \end{aligned}$$

Equations (9) and (11) state that the particle derivative $\frac{\text {d}\mathcal {G}}{\text {d}t}$ can be considered as the time derivative of the function $\mathcal {G}(t)$, but the same cannot be assumed of the material derivative in general since the skeleton and the fluid may undergo different motions.

*Conservation of mass* The conservation of fluid mass can be considered by posing (7) in terms of mass density $\rho ^f$

12 $$\begin{aligned} \rho ^{f}\phi \text {d}\varOmega = \left( \rho _o^{f}\phi _o + m(\mathbf {X},t) \right) \mathrm{d}\varOmega _o \end{aligned}$$

where *m* is defined as the current additional fluid mass content per unit reference volume $\text {d}\varOmega _o$, above the initial level. If a distributed source $s(\mathbf {x},t) = S(\mathbf {X},t)$ is present,

13 $$\begin{aligned} \frac{\text {D}\mathscr {G}}{\text {D}t} = \int _{\varOmega _o} S \mathrm{d}\varOmega _o. \end{aligned}$$

Applying (11) to the above leads to the Lagrangian fluid continuity equation

14 $$\begin{aligned} \frac{\text {d}m}{\text {d}t} + \nabla _{\mathbf {X}}\cdot \bigl (\rho ^f \mathbf {W}\bigr ) = S. \end{aligned}$$

In the absence of solid phase mass creation, the balance of the skeleton mass is simply

15 $$\begin{aligned} \rho ^s(1-\phi ) \text {d}\varOmega = \rho ^s_o(1-\phi _o) \text {d}\varOmega _o. \end{aligned}$$

*Equation of motion* The conservation of momentum in the porous medium is often examined for the whole mixture, since the balance within an individual phase gives rise to a distributed interaction force term which must be additionally defined. For brevity, we refer the reader to a derivation elsewhere (Coussy 1995) and focus instead on describing the results. The Lagrangian equation of motion reads

16 $$\begin{aligned} \nabla _{\mathbf {X}}\cdot \left( {\mathsf {FS}}\right) + m^s(\mathbf {f}-\mathbf {a}^s) + m^f(\mathbf {f}-\mathbf {a}^f) = 0 \end{aligned}$$

where ${\mathsf {S}}, \mathbf {f}$ and $\mathbf {a}$ represent the second Piola–Kirchoff stress, body force density and acceleration, respectively. The two density-like quantities $m^s$ and $m^f$ denote, respectively, the skeletal and fluid mass content per unit reference volume $\mathrm{d}\varOmega _o$, such that

17 $$\begin{aligned} m^s = \rho ^s J(1-\phi ), \qquad m^f = \rho ^{f}_o\phi _o + m \end{aligned}$$

noting that $m^s$ remains constant and equal to the reference $m^s_o=\rho _o^s(1-\phi _o)$ by virtue of (15).

*Stress partition* Consistent with the standard theory, the Cauchy stress ${\mathsf {\sigma }}$ is related to ${\mathsf {S}}$ and first Piola–Kirchoff stress ${\mathsf {P}}$ via

18 $$\begin{aligned} \sigma = \frac{1}{J}{\mathsf {FSF}}^T = \frac{1}{J}{\mathsf {PF}}^T \end{aligned}$$

where the total stress in the macroscopic medium is the sum of individual phase stresses $({\mathsf {\sigma }}={\mathsf {\sigma }}^S+{\mathsf {\sigma }}^F)$, which in turn, is posited to be the volume-weighted average of the actual stress in the microscopic constituent. Consequent investigations with micro–macro averaging technique has confirmed the correspondence between the integral of microscopic free energy potential with the macroscopic stresses (Buhan et al. 1998), and derived macroscopic equation of motion from the microscopic momentum balance (Coussy 2004).

The fluid stress tensor can be expressed as ${\mathsf {\sigma }}^f = -p {\mathsf {I}} + {\mathsf {\tau }}$, where *p* is termed *pore pressure* and ${\mathsf {\tau }}$ represents the fluid shear stress. In practice, the shear term is often disregarded. In the coronary circulation, we assume that such a contribution is secondary to the drag at the internal walls (the main mechanism behind the Darcy flow; see below), and adopt ${\mathsf {\sigma }}^{f} = -p {\mathsf {I}}$ in the following, including also for the one-dimensional flow formulation. Lastly, we remark that this hydrostatic stress state inherently embeds a constitutive assumption, and a specific formulation should be posed consistently.

*Darcy’s law* The pore fluid motion is modelled by Darcy’s law, which is a linear conduction law in the form

19 $$\begin{aligned} \mathbf {w} = -{\mathsf {K}} \nabla _{\mathbf {x}}p. \end{aligned}$$

Note that here the effects of gravity, fluid inertia or shear (Brinkman correction) are ignored, since in the coronary perfusion, we regard such terms to be of secondary importance. In general, the permeability K is a tensor. To ensure that a pressure gradient accelerates the flow in a consistent direction, K must be positive definite. The phenomenological foundations underlying Darcy’s law has been consolidated through subsequent micro–macro averaging derivation, which arrived at the identical expression by assuming Stoke’s law and fluid incompressibility in a general porous medium without discontinuity (Whitaker 1986).

As the pores deform, the permeability of the medium will also undergo changes. By considering the Poiseuille conductance *C* of a representative cylindrical vessel, we write

20 $$\begin{aligned} C \propto \frac{r^4}{l} \propto \frac{a^2}{l} = \frac{v^2}{l^3} \end{aligned}$$

where *l*, *r*, *a* and *v* correspond to the length, radius, area and volume of the segment. Upon changes to the vessel volume,

21 $$\begin{aligned} \frac{C}{C_o} \propto \frac{{}^{v^2}/_{l^3}}{{}^{v_o^2}/_{l_o^3}}. \end{aligned}$$

If we assume that the change in segment length is negligible, (21) becomes

22 $$\begin{aligned} \frac{C}{C_o} \propto \frac{v^2}{v_o^2} = \frac{|\varOmega ^f|^2}{|\varOmega _o^f|^2} = \biggl (\frac{J\phi }{\phi _o}\biggl )^2. \end{aligned}$$

If, on the other hand, isotropic permeability is assumed, $l = \root 3 \of {J}\,\,l_o$ in three dimensions such that

23 $$\begin{aligned} \frac{C}{C_o} \propto J\frac{\phi ^2}{\phi _o^2}, \quad \therefore {\mathsf {K}} = \biggl (J\frac{\phi ^2}{\phi _o^2}\biggl ) {\mathsf {K}}_o. \end{aligned}$$

Equations (22) and (23) represent the upper and lower bounds on ${\mathsf {K}}$ scaling.

#### Constitutive law

This section details the development of a specific constitutive law applied in numerical simulations (Sect. 3). The formulation is intended for cardiac tissue, for which both the solid and fluid phases can reasonably be treated as incompressible.

*Thermodynamic perspectives on constitutive law* In a continuum, the second law of thermodynamics takes the form of Clausius–Duhem inequality. The general expression for a porous medium has been previously derived (Coussy 1989; Dormieux and Stolz 1992). In the isothermal regime, the intrinsic dissipation *D* is

24 $$\begin{aligned} D = {\mathsf {S}}:\frac{\text {d}{\mathsf {E}}}{\text {d}t} + p \frac{\text {d}(J\phi )}{\text {d}t} - \frac{\text {d}\varPsi ^s}{\text {d}t} \ge 0 \end{aligned}$$

where $\varPsi ^s$ is the (Lagrangian) free energy density of the skeleton. Note that $p\frac{\text {d}(J\phi )}{\text {d}t}$ represents the strain work performed by action of the pore pressure on the skeleton via the internal walls. It is observed that in the absence of this term, we would recover the dissipation of a standard solid involving only the strain work rate ${\mathsf {S}}:\frac{\text {d}{\mathsf {E}}}{\text {d}t}$. The term $J\phi = \frac{\text {d}\varOmega ^f}{\text {d}\varOmega _o}$ is known as the Lagrangian porosity, which measures the current fluid content per unit reference volume. While on heuristic grounds, one may expect $\phi \,\,(=\frac{\text {d}\varOmega ^f}{\text {d}\varOmega })$ to be associated with the fluid action, because fluid inflow will, in general, lead to an associated change in $\text {d}\varOmega , J\phi$ offers a more appropriate account of the fluid work rate. Thus (24) shows that *p* is the thermodynamic force driving the change in $J\phi$.

*Poroelasticity* is characterised by zero intrinsic dissipation in the system. Hence, putting $D=0$ leads to the state equations

25 $$\begin{aligned} {\mathsf {S}} = \frac{\partial \varPsi ^s}{\partial {\mathsf {E}}}, \quad p=\frac{\partial \varPsi ^s}{\partial (J\phi )}. \end{aligned}$$

Note that inherent in (25) is an assumption of normality between the state variables E and $J\phi$, that is, variation of either particular state can occur independently of the other variable. This property is demonstrated by materials with compressible microscopic solid constituent, whereby the pore volume can change in the absence of macroscopic deformation.

*Matrix incompressibility*

The altered state of the skeletal stress under solid matrix incompressibility can be examined by adapting the dissipation expression appropriately. By putting $\rho ^s = \rho _o^s$, Eq. (15) can be written as

26 $$\begin{aligned} J - J\phi \,\, = \,\, 1-\phi _o \,\, = \,\, \phi _o^s \end{aligned}$$

such that $\frac{\text {d}J}{\text {d}t} = \frac{\text {d}(J\phi )}{\text {d}t}$. Now using the volume change rate identity (Bonet and Wood 2008, Chpt4)

27 $$\begin{aligned} \frac{\text {d}J}{\text {d}t} = J{\mathsf {C}}^{-1}:\frac{\mathrm{d}{\mathsf {E}}}{\mathrm{d}t} \end{aligned}$$

Equation (24) can be re-expressed as

28 $$\begin{aligned} D' = \left( {\mathsf {S}}+pJ{\mathsf {C}}^{-1}\right) :\frac{\text {d}{\mathsf {E}}}{\text {d}t} - \frac{\text {d}\varPsi ^{s'}}{\text {d}t} \ge 0. \end{aligned}$$

The new expression shows that the physically pertinent stress in the medium is no longer the total stress S, but rather the component excluding pore pressure

29 $$\begin{aligned} {\mathsf {S}}' = {\mathsf {S}} + pJ{\mathsf {C}}^{-1} \end{aligned}$$

which is classically referred to as the *effective* stress. It also shows that $J\phi$ is no longer a state variable, thus reducing (25) to simply

30 $$\begin{aligned} {\mathsf {S}}'=\frac{\partial \varPsi ^{s'}}{\partial {\mathsf {E}}}. \end{aligned}$$

In other words, the knowledge of *p* or $J\phi$ is no longer required to determine the skeletal free energy, as the actual work performed by the fluid on the skeleton via internal walls will be self-evident from the observed deformation F by virtue of the constraint (26). Note however, in contrast to the hyperelastic incompressibility, the new constitutive equation (30) does not have to be posed in terms of the strictly distortional component of the strain tensor. In fact, it would be undesirable to do so in the current context, as it would imply zero resistance against volumetric dilation. The difference arises from the fact that matrix incompressibility does not imply skeletal incompressibility—through fluid inflow, macroscopic volume change is still possible, thus the macroscopic medium must be regarded as compressible.

*Method of Lagrange multiplier*Although the definition of effective stress reveals physical insights, (30) leaves pore pressure indeterminate, and the solution process must devise a way to calculate consistent fluid stress states. For materials with nearly incompressible matrix, a procedure described in Chapelle and Moireau (2014) may be employed. In this work, the incompressibility of the material is addressed by the method of Lagrange multiplier. First, the general free energy expression admitting matrix compressibility effects is augmented with the constraint (26)

31 $$\begin{aligned} \widetilde{\varPsi }^s = \varPsi ^s - \lambda (J-J\phi -1+\phi _o) \end{aligned}$$

where the superscript $(\sim )$ denotes a constrained quantity. It follows from (25) that

32 $$\begin{aligned}&\widetilde{p} = \frac{\partial \varPsi ^s}{\partial (J\phi )} + \lambda \end{aligned}$$

33 $$\begin{aligned}&\widetilde{{\mathsf {S}}} = \frac{\partial \varPsi ^s}{\partial {\mathsf {E}}} -\lambda \frac{\partial J}{\partial {\mathsf {E}}} = \frac{\partial \varPsi ^s}{\partial {\mathsf {E}}} + \frac{\partial \varPsi ^s}{\partial (J\phi )}\frac{\partial J}{\partial {\mathsf {E}}} - pJ{\mathsf {C}}^{-1} \end{aligned}$$

where (33) was expanded using (32). Upon comparison with (29), the effective stress is identified as

34 $$\begin{aligned} {\mathsf {S}}' = \frac{\partial \varPsi ^s}{\partial {\mathsf {E}}} + \frac{\partial \varPsi ^s}{\partial (J\phi )}\frac{\partial J}{\partial {\mathsf {E}}} \end{aligned}$$

which can be shown to be consistent with (30) if we consider $J\phi$ as a function of E such that $\varPsi ^{s'}=\varPsi ^{s'}({\mathsf {E}},J\phi ({\mathsf {E}}))$, and the equivalence in rates of *J* and $J\phi$ due to (26) upon fulfilment of the constraint.

*Compaction limit*For a physically sensible behaviour, the constitutive law must appropriately address the limit cases, which occur when the porosity reaches a value of 0 or 1. As pointed out in Chapelle et al. (2010), Eq. (26) guarantees $1-\phi >0$ for $0 < J < \infty$; therefore, no explicit measures are required to ensure $\phi <1$. Against the compaction limit $(\phi =0)$, a barrier potential previously proposed by Federico and Grillo (2012) is considered here. While the purpose of the original formulation was to control the bulk modulus in the solid skeleton, unnecessary in the current work due to the Lagrange multiplier approach, it has several desirable properties which are exploited here to modify the pore pressure characteristics. In particular, the adapted potential $\varTheta$ remains inactive until compaction is approached (in that it contributes zero pressure and zero elastance at non-negative volumetric strain), but when active, tends towards infinite pressure and elastance, which can be used to prevent further fluid extraction. These conditions can be expressed as

35 $$\begin{aligned}&\frac{\partial \varTheta }{\partial (J\phi )} = \frac{\partial ^2\varTheta }{\partial (J\phi )^2} = 0, \quad \,\, \text {for} \quad J\ge 1, \end{aligned}$$

36 $$\begin{aligned}&-\frac{\partial \varTheta }{\partial (J\phi )}\rightarrow + \infty , \quad \,\, \text {for} \quad J\phi \rightarrow 0, \end{aligned}$$

37 $$\begin{aligned}&\frac{\partial ^2\varTheta }{\partial (J\phi )^2}\rightarrow + \infty , \quad \text {for} \quad J\phi \rightarrow 0. \end{aligned}$$

and the barrier function reads

38 $$\begin{aligned} \varTheta (J\phi ) = \mathscr {H}(\phi _\mathrm{crit}-J\phi )(J\phi -\phi _\mathrm{crit})^{2q}(J\phi )^{-r} \end{aligned}$$

where $\mathscr {H}$ denotes the Heaviside step function which ensures that (38) becomes active only when $J\phi <\phi _\mathrm{crit}$, where $\phi _\mathrm{crit}\in (0,\phi _o]$. The parameter *r* is selected within (0, 1], and *q* is a positive integer. Given these choices, (38) will satisfy (35)–(37).

*Free energy composition*Based on the above developments, we now compose a specific constitutive law. The general form of the free energy has been built up to this point as

39 $$\begin{aligned} \varPsi ^s = \varPhi ({\mathsf {E}},J\phi ) + \varTheta (J\phi ) \end{aligned}$$

where $\varPhi$ fully characterises the behaviour of the porous material above the compaction limit. To facilitate parameter decoupling, we further propose a splitting of this potential such that

40 $$\begin{aligned} \varPsi ^s = {\bar{\varPhi }}({\mathsf {E}}) + {\hat{\varPhi }}(J\phi ) + \varTheta (J\phi ). \end{aligned}$$

Such a decomposition permits the direct incorporation of previous (separate) models of cardiac constitutive and coronary pressure–volume relations.

On the other hand, the response of the macroscopic skeleton depends on the specific composition of its constituent phases. In the simplest case, this can be represented by the initial volume fractions $\phi _o$ and $\phi _o^s({=}1-\phi _o)$ in the free energy expression, to capture correct proportional contribution of the skeleton to the total energy density. Considering the extreme cases, when $\phi _o^s \rightarrow 0$, we would expect ${\mathsf {S}}$ to vanish. Likewise, $\phi _o \rightarrow 0$ will recover a purely solid material. In between these bounds, it can be expected that the lower the initial porosity, the greater the pressure required will be in order to raise the fluid content of the medium by a given amount. A simple modification to (40) can satisfy these conditions

41 $$\begin{aligned} \varPsi ^s = \phi _o^s {\bar{\varPhi }}({\mathsf {E}}) + \frac{1}{\phi _o} \left[ {\hat{\varPhi }}(J\phi ) + \varTheta (J\phi ) \right] . \end{aligned}$$

Due to the free energy being a linear summation, these new scaling terms can be absorbed into the constitutive parameters of the form (40). However, (41) serves to demonstrate that these parameters reflect the volume fractions of the phases at the reference state.

For the specific form of ${\bar{\varPhi }}$, an existing cardiac constitutive law can be employed. Here, we select the structurally based law of Holzapfel and Ogden (2009), which encompasses the preceding orthotropic-type laws (and their various simplifications). For $\hat{\varPhi }$, we adopt the experimentally characterised pressure–volume relation of Bruinsma et al. (1988),

42 $$\begin{aligned} {\hat{\varPhi }}(J\phi ) = \frac{q_1}{q_3}\text {exp}(q_3J\phi ) + q_2J\phi \bigl [ \text {ln}(q_3J\phi )-1 \bigr ] -p_oJ\phi \end{aligned}$$

leading to

43 $$\begin{aligned} \frac{\partial \varPsi ^s}{\partial (J\phi )} = q_1 \text {exp}(q_3J\phi ) + q_2\text {ln}(q_3J\phi ) - p_o \end{aligned}$$

where the constant $p_o$ exists to satisfy the condition $p=0$ at rest volume $(J\phi =\phi _o)$. In (43), the exponential term dominates during large net mass inflow, while the log term dominates during small or negative mass inflow.

*Active stress*For active stress generation we adopt a modified form of a previously proposed model (Kerckhoffs et al. 2003) of the form

44 $$\begin{aligned} \sigma _\mathrm{act}&= T_0\,\varphi \,\, \tanh ^2\left( \frac{t_c}{t_r}\right) \tanh ^2\left( \frac{t_\mathrm{max}-t_c}{t_d}\right) \end{aligned}$$

where

45 $$\begin{aligned} \varphi&= \tanh \left( a_6(\lambda -a_7) \right) , \end{aligned}$$

46 $$\begin{aligned} t_r&= t_{r0} + a_4\left( 1-\varphi \right) \end{aligned}$$

and $t_c = \text {mod}(t,t_\mathrm{period})$. Here, $T_0, t_\mathrm{max}$ and $\lambda$ denote the peak stress scaling parameter, activation duration and fibre stretch ratio, respectively. $\sigma _\mathrm{act}$ is assigned as an additional component in the total stress tensor.

### One-dimensional vascular flow

#### Network flow model

The one-dimensional model of vascular flow has been well established in the literature (see Lee and Smith (2012) and Vosse and Stergiopulos (2011) for review). We follow the formulation employed in previous work (Lee and Smith 2008) with conservation equations

47 $$\begin{aligned}&\frac{\partial }{\partial t}\begin{bmatrix} A \\ Q \end{bmatrix} + \frac{\partial }{\partial x} \begin{bmatrix} Q \\ \alpha \frac{Q^2}{A} + \int c^2 dA \end{bmatrix} = \begin{bmatrix}0 \\ -K \frac{Q}{A} \end{bmatrix} \end{aligned}$$

where *A* and *Q* denote the cross-sectional average area and flow rate, *K* represents the friction coefficient at the lumen wall, which can be written as (Smith et al. 2002)

48 $$\begin{aligned} K = \frac{2\pi \alpha \nu }{\alpha -1}. \end{aligned}$$

The parameter $\alpha$ controls the shape of the flow profile across the cross section of the vessel.

The derivation of the above system involves posing a pressure–area relation

49 $$\begin{aligned} p = \beta \bigl ( \sqrt{A} - \sqrt{A_o} \bigr ) \end{aligned}$$

which leads to the distensibility *D* and wave speed *c*

50 $$\begin{aligned} D = \frac{2}{\beta \sqrt{A}}, \quad c = \frac{1}{\sqrt{\rho D}} = \sqrt{\frac{\beta }{2\rho }}A^{\frac{1}{4}} \end{aligned}$$

showing that vascular wave speed is proportional to the square root of wall stiffness parameter $\beta$. The junction equations include the conservation of mass and total pressure and the compatibility relations as presented in Lee and Smith (2008).

#### Coupling with porous domain

The geometrical interface between the explicit vascular and porous flow regimes within the modelling framework is represented by the meso-scale vessels which progressively bifurcate into smaller segments forming a distributed network. A simplified treatment of this transition zone as a point source in the porous domain is undesirable, since localised outflow will lead to the development of unphysiological pressure and velocity peaks. Therefore, we assume that the exchange of fluid between a terminal vessel and the porous tissue occurs within a volume $\varOmega _\mathrm{int}$ surrounding the distal end of the vessel $\mathbf {x}_\mathrm{term}$, such that

51 $$\begin{aligned} \rho ^f Q_{1D}(t) = \frac{1}{|\varOmega _\mathrm{int}|} \int _{\varOmega _\mathrm{int}} S(\mathbf {x},t) \mathrm{d}\varOmega \end{aligned}$$

where, for clarity, the variables associated with the one-dimensional vascular domain are denoted by the subscript 1*D* and *S* denotes a distributed source in the porous domain. Furthermore, we express *S* via a distribution function *f*

52 $$\begin{aligned} S(\mathbf {x},t) = \rho ^f Q_{1D}(t) f(\mathbf {x}-\mathbf {x}_\mathrm{term}), \end{aligned}$$

which, together with (51) implies

53 $$\begin{aligned} \frac{1}{|\varOmega _\mathrm{int}|} \int _{\varOmega _\mathrm{int}} f(\mathbf {x}-\mathbf {x}_\mathrm{term}) \mathrm{d}\varOmega = 1. \end{aligned}$$

In the absence of detailed anatomical information, we approximate *f* with a Gaussian function. The pressure–flow relationship of the coupling interface can be established by regarding the meso-scale vessels as a predominantly resistive element such that

54 $$\begin{aligned} Q_{1D}(t) = \frac{p_{1D}(t)-\bar{p}(t)}{R_\mathrm{term}} \end{aligned}$$

where, as a first approximation, we define the average pressure $\bar{p}(t)$ as

55 $$\begin{aligned} {\bar{p}}(t) = \frac{1}{|\varOmega _\mathrm{int}|} \int _{\varOmega _\mathrm{int}} p(\mathbf {x},t) f(\mathbf {x} - \mathbf {x}_\mathrm{term}) \mathrm{d}\varOmega . \end{aligned}$$

### Systemic haemodynamics

As is common in the literature, we employ a windkessel-type systemic boundary conditions to the ventricular model. The aortic valve dynamics has been previously characterised by Korakianitis and Shi (2006), based on an orifice model accounting for leaflet angular position. However, the original formulation allowed instantaneous changes in the aortic valve flow, which resulted in rapid fluctuations of pressure following the isovolumic phases. To address this issue, the valve flow dynamics has been altered to be a first-order ODE such that

56 $$\begin{aligned}&\frac{\text {d}Q_{ao}}{\text {d}t} = \frac{1}{T_{ao}}\left( Q_{ss} - Q_{ao} \right) \end{aligned}$$

57 $$\begin{aligned}&Q_{ss} = \left\{ \begin{array}{lll} CQ_{ao} AR_{ao} \sqrt{P_{lv} - P_{as}}, &{}\quad {P_{lv} \ge P_{as}} \\ -CQ_{ao} AR_{ao} \sqrt{P_{as} - P_{lv}}, &{}\quad {P_{as} > P_{lv}} \end{array}\right. \end{aligned}$$

58 $$\begin{aligned}&AR_{ao} = \left( \frac{1-\cos (\theta )}{1-\cos (\theta _{max})} \right) ^2 \end{aligned}$$

59 $$\begin{aligned}&\frac{\text {d}^2\theta }{\text {d}t^2} = K_{1ao} (P_{lv}-P_{as}) \cos (\theta ) - K_{fao}\frac{\text {d}\theta }{\text {d}t} \end{aligned}$$

where $\theta , P_{as}$ and $P_{lv}$ denote the valve angular position, aortic pressure and LV pressure, respectively. The systemic boundary condition is modelled with a three-element windkessel, which had been shown to accurately reproduce physiological systemic impedance (Westerhof et al. 2009)

60 $$\begin{aligned} P_{as}&= P_s + R_a Q_{ao} \end{aligned}$$

61 $$\begin{aligned} \frac{\text {d}P_s}{\text {d}t}&= \frac{1}{C}\left[ Q_{ao}-\frac{P_s}{R_s} \right] \end{aligned}$$

where $P_s$ represents the lumped systemic pressure. The flow through the mitral valve is modelled as

62 $$\begin{aligned} Q_{mi} = \left\{ \begin{array}{l@{\quad }l} CQ_{mi}\, \tanh \left( -C_{mi}\, (P_{lv}-P_{la})\right) , &{} P_{la}>P_{lv} \\ 0, &{} P_{la}\le P_{lv}. \end{array}\right. \end{aligned}$$

The coupling between this system of equations and the ventricular model is achieved through the cavity pressure $P_{lv}$, which is induced by the myocardial contraction and set in (59), and $Q_{ao}$ which is updated by (56) and imposed on the deformation through application of the following constraint to the finite element problem using a Lagrange multiplier

63 $$\begin{aligned} \int _{\partial \Gamma } \frac{\text {d}\mathbf {U}}{\text {d}t}\cdot \mathbf {n} = -Q_{ao} \end{aligned}$$

where $\partial \Gamma$ denotes the endocardial surface.

### Solution procedures

The numerical solution of the coupled model is accomplished using CHeart, an mpi-based multi-physics finite element solver developed at King’s College London (Lee et al. 2016). Due to the disparate parallel computational requirements of each subproblem, we employ a sequential approach whereby the solution of each coupled system is iterated until a nonlinear system convergence is achieved at each time step. The poroelastic system (14), (16) and (19) was discretised using a Galerkin finite element method with quadratic/linear/linear elements for the displacement/pressure/mass mixed formulation, while the one-dimensional vascular system (47) was discretised using a fifth-order spectral element method with Crank–Nicolson time stepping. Details of mesh construction and refinement are presented in Sect. 3.1. The simulations were executed on the in-house HPC resource (SGI Altix UV 1000). With 128 cores per simulation using a time step size of $0.1\,\text {m}\,\text {s}$, the typical solution wall time was around 25 h.

Further details of the numerical formulation of individual physics can be found elsewhere (coronary flow (Lee and Smith 2008), elasticity (Hadjicharalambous et al. 2014) and porous flow in large deformation (Cookson et al. 2012)).

**Fig. 2:**
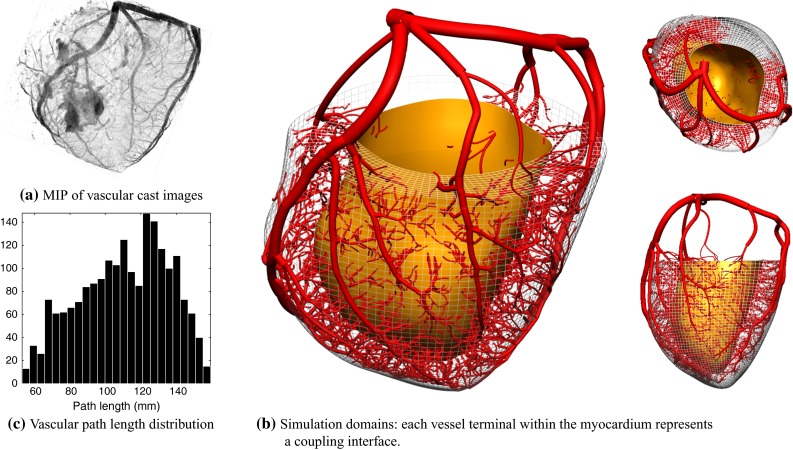
The computational simulation meshes were created from 50-$\upmu$m-resolution 3D image stacks of porcine myocardium and coronary vasculature. A maximum intensity projection (MIP) of the vascular images is shown in **a**. The myocardial mesh was truncated near the valve plane, and vascular network was modified to achieve an even distribution of terminal segments throughout the myocardium (see text for details). The distribution of lengths from vascular root to each distal terminal node $(11\pm 2.5$ cm) is shown in **c**

## Results

### Geometrical model construction

The image-to-model generation process is briefly outlined here and summarised in Fig. 2. The heart and coronary geometries were obtained from a previous experimental study performed on a 40-kg Danish Landrace pig (Schuster et al. 2010). After injection with a fluorescent vascular casting polymer in the left main stem, the heart was frozen in an unloaded state and imaged using a cryomicrotome multichannel acquisition at $50\,\upmu$m resolution (Spaan et al. 2005). Subsequently, the myocardial geometry was segmented, and a custom template mesh fitting procedure was employed. The right ventricular wall and papillary muscles were excluded, as is customary in clinical perfusion analysis.

**Table 1:** Baseline parameters for the systemic, haemodynamic and contractile models

Systemic circulation parameters			Coronary haemodynamic parameters			Active stress parameters		
$\theta _\mathrm{max}$	75	$^\circ$	$\rho$	$1.05\times 10^{-6}$	$\text {kg}\,\text {mm}^{-3}$	$a_4$	3.2	s
$T_{ao}$	0.01	*s*	$\mu$	$3.36\times 10^{-6}$	kPa s	$a_6$	2.0	Dimensionless
$CQ_{ao}$	$9.59\times 10^{5}$	$\text {mm}^3/(\text {s}\,\text {kPa}^{0.5})$	$\alpha$	1.05	Dimensionless	$a_7$	0.7	Dimensionless
$K_{1ao}$	750	$(\text {kPa}\,\text {s})^{-1}$	*c*	15,000	$\text {mm}\,\text {s}^{-1}$	$T_0$	15,000	kPa
$K_{fao}$	50	$\text {s}^{-1}$	$q_1$	0.022	$\text {kPa}$	$t_{r0}$	0.16	s
$R_a$	$1.2\times 10^{-5}$	$\text {kPa}\,\text {s}/\text {mm}^3$	$q_2$	1.009	kPa	$t_d$	0.03	s
$R_s$	$2.2\times 10^{-4}$	$\text {kPa}\,\text {s}/\text {mm}^3$	$q_3$	80	Dimensionless	$t_\mathrm{max}$	0.3	s
*C*	7000	$\text {mm}^3/\text {kPa}$	$\phi _o$	0.06	Dimensionless	QRSd	0.06	s
$CQ_{mi}$	$2.0\times 10^5$	$\text {mm}^3/s$	$\gamma$	$1.0\times 10^{-7}$	$\text {mm}^3\,\text {s}^{-1}\,\text {kPa}$			
$C_{mi}$	37.5	$(\text {kPa})^{-1}$	$p_\mathrm{drain}$	0.5	kPa			

The vascular images were reconstructed as described in Goyal et al. (2013) to full detail. The resulting mesh was then truncated to restrict its extent to the upper arterial network, by dividing the myocardium into approximately 0.02 mL volumes and seeking a supplying vessel segment within each, with a target diameter of $200\,\upmu$m. These seeded segments were traced back up to the root and the set of all traversed segments comprised the simulation domain. This ensured an even distribution of vascular termini throughout the myocardium. Due to there being no polymer injected in the right coronary vessel, the inferoseptal and parts of the anteroseptal wall lack vasculature. In addition, vessel segments were assumed to be of uniform unstressed area, as the resolution of the imaging system did not permit a reliable characterisation of the tapering behaviour in all segments. The resulting truncated mesh has an LV volume of 40.6 mL, unloaded LV cavity volume 48.5 mL, and 3910 vascular segments with 1990 distal termini totalling 2.4 mL excluding the microcirculation. The distribution of path lengths from the root to each terminal ($11\pm 2.5$ cm) is shown in Fig. 2c.

The computational mesh for the poroelastic problem was generated using quadratic/linear hexahedral elements, yielding around 30,000 degrees of freedom. The vascular mesh was refined to yield around 187,000 degrees of freedom, which was found to be necessary to resolve the accurate wave propagation behaviour as indicated by a mesh refinement study.

### Physiological baseline conditions

*Parameters* The physiological baseline parameters were largely adopted from the literature. The passive myocardial parameters were obtained from Holzapfel and Ogden (2009), for which a downscaling was necessary to tune the diastolic behaviour. A uniform factor of 0.25 was applied to the parameter set. In the absence of subject-specific fibre measurement, a linear endo-to-epi distribution of fibre angles from $+60^{\circ }$ to $-60^{\circ }$ was used (Zhang et al. 2013). For the active tension model, adjustments were made to $t_{r0}, t_d$ and $T_0$ to achieve a sufficiently fast relaxation rate. For clarity in coronary wave interpretation, a simple linear activation wave spreading from endocardium to epicardium lasting 60$\,\text {m}\,\text {s}$ was prescribed without a base–apex gradient.

The permeability ${\mathsf {K}}$ of the porous domain was assumed to be isotropic. Although no porcine-specific data was available, a recent study on the capillary network of rat myocardium has estimated the principal component of the permeability tensor to be on the order of $3\times 10^{-3}\text {mm}^3\,\text {s}\,\text {kg}^{-1}$ (Smith et al. 2014). Using the ratio between reported capillary densities in pig (Koudstaal et al. 2013) and rat (Kerckhoven et al. 2004) myocardium, the permeability was adjusted to be $2\times 10^{-3}\text {mm}^3\text {s}\,\text {kg}^{-1}$. The porous constitutive parameters were based on the capillary compartment parameters in Bruinsma et al. (1988), but $q_3$, which scales the volume response, was adjusted to include a partial influence from the larger, arteriolar microvessels. As only a single porous compartment was used in this model, the role of the porous domain should be considered to represent the combined behaviour of both arteriolar and capillary microcirculation.

The elastic properties $\beta$ of the vessel segments were determined by assuming a constant uniform wave speed of $15\,\hbox {ms}^{-1}$, thus implying a constant distensibility. Currently, there is no gold standard technique for measuring local coronary pulse wave velocity, and inter-subject variations ranging from $11\, \text {m}\,\text {s}^{-1}$ (Aguado-Sierra et al. 2006) to above $20\,\text {m}\,\text {s}^{-1}$ (Rivolo et al. 2014) have been reported in pigs. All other parameters were determined by manual tuning. The complete set of baseline parameters are listed in Table 1.

**Fig. 3:**
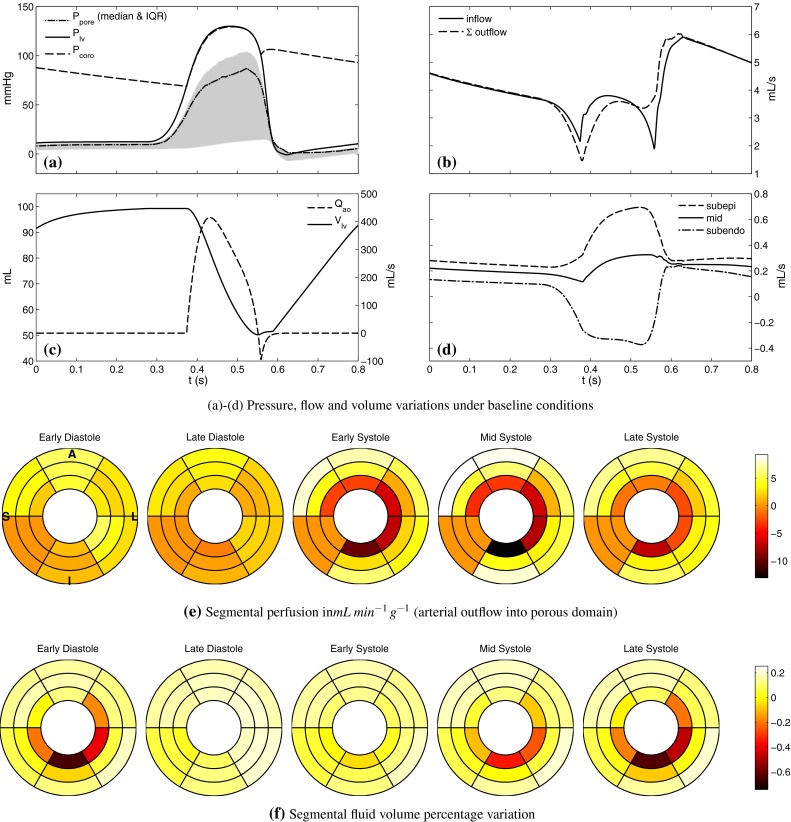
Baseline results **a** coronary inlet pressure, LV cavity pressure and distal pore pressure (median and inter-quartile range over the nodes). Although not shown in this figure, the maximum pore pressure in excess of LV pressure was found during systole, consistent with experimental observations. **b** Inflow and total outflow of the upper arterial vascular network. Inflow exceeds the outflow in early systole as the influence of cardiac contraction is greater on the distal arterial flow. The reverse happens during late systole, as the stored flow is discharged. **c** LV cavity volume and aortic outflow. Transient reversal in flow is observed at end systole, enabled by the modified valve dynamics (see text for details). **d** Coronary flow across transmural layers shows an augmented systolic flow in the subepicardial layer and a reversed flow in the subendocardial layer. **e** Tissue segmental perfusion in the mid-equatorial portion of myocardium. Systolic endo-to-epi fluid shift caused by increased pressure can clearly be seen. The inferoseptal segment receives zero flow, due to a lack of vasculature in the region. Slice orientation is shown on the first diagram (anterior/inferior, lateral/septal). **f** Variation of blood volume in the tissue as a percentage of total reference material volume. Around 1 % maximum variation is observed in the baseline conditions. The deficit in the subendocardial layer lags perfusion rate in time due to capacitance effects

*Boundary conditions* To prevent free body translation of the ventricle, the epicardial ring of nodes at the base was fixed in the longitudinal direction; however, the base plane surface was permitted to deform under a Maxwell-type constraint. This was necessary to prevent large unphysiological hydrostatic pressures developing in the basal region which could adversely affect the porous flow.

The lateral component of translation was restricted by a linear-spring-type model applied to the epicardium which penalised displacement in the surface-normal direction.

The pressure at the inlet of the vascular network was prescribed to be equal to the aortic sinus pressure $(p_{as})$ determined by the systemic windkessel model. The outflow conditions from the porous domain was prescribed as a variable resistor of the form

64 $$\begin{aligned} Q_\mathrm{out} = \gamma (J,\phi ) (p_\mathrm{pore} - p_\mathrm{drain}) \end{aligned}$$

where $\gamma$ was subject to the same scaling as the permeability ${\mathsf {K}}$ as in (23).

*Baseline function* The results of the baseline simulation are shown in Fig. 3. In addition, the bull’s eye diagrams in Fig. 3e, f depict segmental perfusion (estimated by the sum of coronary outflow from the vascular mesh) and fluid mass accumulation (*m*) in the mid-equatorial ring with three transmural layers. The global functional measures are as follows: ejection fraction 49.5 %, perfusion 6.2$\,\text {mL}\,\text {g}^{-1}\,\text {min}^{-1}$, perfusion pressure 129/69$\,\text {mmHg}$, diastolic time fraction 63 %.

### Wave intensity analysis

Wave intensity $(\text {d}I)$ is defined as the product of the increments in pressure $(\text {d}p)$ and velocity $(\text {d}v)$ during a small time interval $(\text {d}t)$. The theory of WIA allows the total intensity to be separated into components according to their direction of travel (denoting forward as proximal-to-distal) and classified according to their pushing/compression (increasing pressure) or suction/expansion effects on pressure (Parker 2009). As mechanical disturbance on vessels can result in a propagating wave, coronary wave dynamics are sensitive to the events taking place in the myocardial environment surrounding the vasculature, as well as those internal to the vessel network.

The application of WIA in the human coronary circulation identified six major waves and the associated cardiac events from which they originate (Davies et al. 2006). Of these, the dominant backward-travelling suction wave has since received the most attention, as it signals the forward acceleration of arterial inflow in diastole, during which around 80 % of anterograde flow occurs. The source of this wave has been attributed to the ventricular relaxation which eases the compressive forces acting on the small vessels within the myocardium. In addition, the dominant forward-travelling pushing wave originating from early ventricular ejection has been shown to carry an equivalent magnitude of *cumulative wave intensity* (also referred to as wave energy, wave area or the time integral of wave intensity (Siebes et al. 2009).

**Fig. 4:**
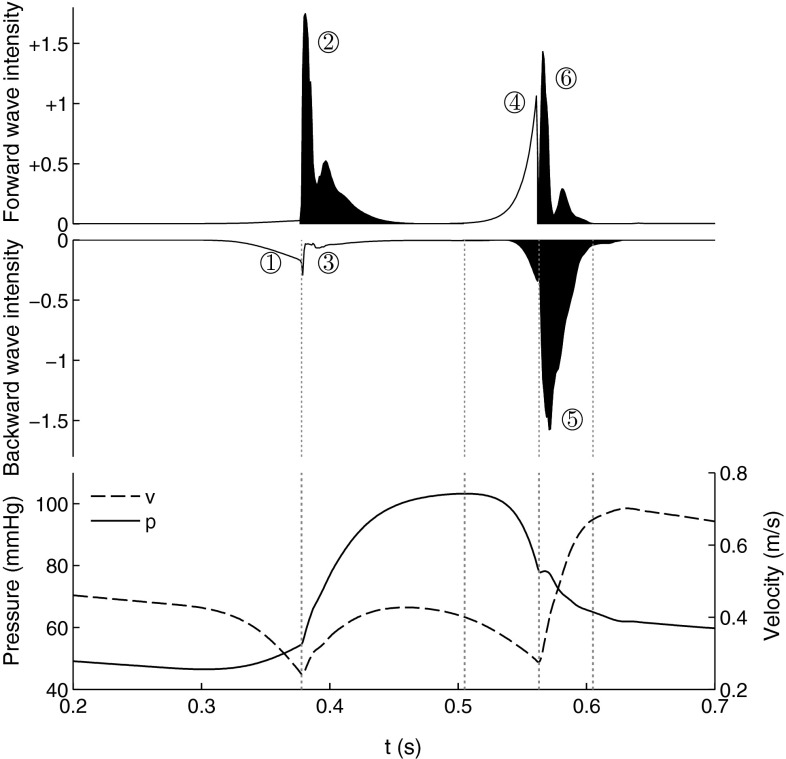
Wave intensity profiles and coronary pressure and velocity calculated from baseline results. The waves associated with anterograde acceleration of flow are coloured in *black*. The six major waves are labelled in accordance with Table  2

The six major waves can be identified in the WI profile calculated from the baseline simulations, in Fig. 4. The WI shape was observed to exhibit a dependence on the measuring location. Reported results were obtained from a location approximately 6 cm along the LAD. The % contributions of the individual cumulative wave intensities compare well with those reported in Davies et al. (2006), as summarised in Table 2.

**Table 2:** Cumulative proportional wave intensity: comparing model results with experimental measurements of Davies et al. (2006)

Wave-type	Experimental %	Model %
① Early backward pushing wave	$1.9\pm 2.1$	5.1
② Dominant forward pushing wave	$22.3\pm 7.9$	27.4
③ Late backward pushing wave	$20.5\pm 2.9$	2.8
④ Forward suction wave	$18.9\pm 4.0$	13.4
⑤ Backward suction wave	$30\pm 5.7$	37.3
⑥ Late forward pushing wave	$6.1\pm 2.4$	14.1

### Wave sensitivity to model parameters

**Fig. 5:**
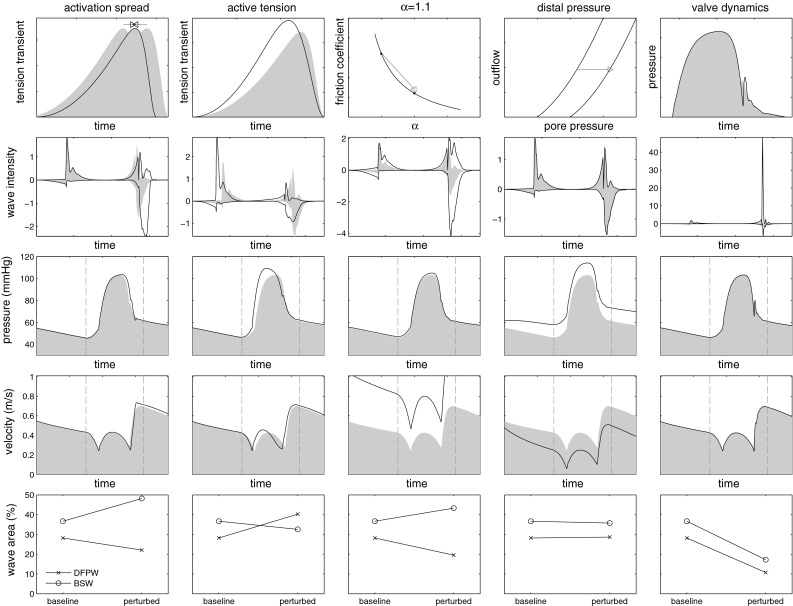
Modified coronary waveforms under parameter perturbation. The applied changes are illustrated in the *top row*, and results of adjusting QRS duration (*first column*), active tension transient (*second column*), vascular resistance (*third column*), distal outflow pressure (*fourth column*) and aortic valve transition rate (*fifth column*) are shown below. The *bottom row* shows the change in % area of dominant forward pushing wave (DFPW) and backward suction wave (BSW). The *grey patches* show the baseline results for comparison. Refer to Sect. 4.2.1 for detailed descriptions

The dependency of wave characteristics on the underlying parameters was investigated through local perturbation around the baseline conditions. Specifically, we examined the effects of contractile (active tension), haemodynamic (viscous resistance, wave speed and outflow), porous (outflow and pressure–volume characteristics) and valve dynamic (rate of transition) parameters on coronary waves. The modified wave intensities and WI indices under the altered parameter sets are summarised in Fig. 5. The top row summarises the perturbation applied to the baseline parameters.

## Discussion

### Baseline parameterisation

The baseline pressure results in Fig. 3a reveal a subtle but important difference between the LV cavity pressure $p_{lv}$ and the microvascular pore pressure $p_\mathrm{pore}$, which is that the peak of the two pressures does not necessarily coincide in time. This is significant because, as wave-generating mechanisms, these pressures act on opposite locations of the coronary network and at times can act in an independent manner. The shape of $p_{lv}$ can be modified through the systemic haemodynamic parameters and under intact aortic valve function $p_{lv}$ is a dominant driving force for the coronary inlet pressure as the figure shows. On the other hand, during systole, $p_\mathrm{pore}$ is governed mainly by the intrinsic myocardial contraction. Therefore, in early systole, the rate of rise of $p_{lv}$ dominates the forward initiated coronary waves, but in late systole, as systemic capacitance begins to become exhausted, $p_{lv}$ stabilises while the intramyocardial stress continues to rise at near-constant LV pressure. Thus, the shape of $p_\mathrm{pore}$ in late systole follows largely that of the tension transient (e.g. see Fig. 5). It is the sharp drop in the intramyocardial stress which enables the generation of the backward suction wave. This mechanism is examined further below in Sect. 4.2.2.

In the literature, several mechanisms have been proposed to characterise the flow impeding forces exerted on coronary vessels by the myocardium, although no single mechanism has been shown to fully explain the experimental observations (Westerhof et al. 2006). In a recent model-based screening investigation, it was suggested that a combination of two forces—shortening-induced intracellular pressure (SIP) and cavity-induced extracellular pressure (CEP)—can qualitatively account for a broad range of existing data (Algranati et al. 2010). This investigation was, however, limited by the phenomenological manner in which each mechanism and their interaction were formulated. CEP was prescribed to be a linear function of the wall depth according to experimental observations; however, the transmural position of vessels was idealised instead of being based on a measured distribution. SIP was prescribed by a simple scaling of the LV volume waveform (Algranati et al. 2010) and applied without variation in local activation timing or fibre architecture. This was replaced by a scaled LV elastance waveform in later work (Mynard et al. 2014) for a more realistic coronary flow. The two pressures were regarded to be independent and linearly summed. While these assumptions assist our intuitive understanding, they can also affect the resulting wave profile in unknown ways. Both SIP and CEP originate from cellular contraction, and their resultant manifestation is subject to force equilibrium under a given set of boundary conditions. Our model accounts for these physical principles through multiscale modelling of conservation laws. With this, we showed from a biophysical basis that the maximal pressure rise from regionally activating myocytes corresponds to late systolic wave features in coronary flow such as the second dip in velocity, while the maximum cavity pressure is associated with the initial dip (Fig. 3a, b).

The total inflow and outflow from the one-dimensional arterial network shown in Fig. 3b reflect another mechanism of interaction, between coronary flow and contraction. While both traces feature a sharp drop during the isovolumic phases, at isovolumic contraction inflow is greater than the outflow, while the trend reverses at isovolumic relaxation. This dynamic is a product of the vascular compliance (both proximal and microvascular), as initially described by the intramyocardial pump model (Spaan 1985). When the total flow is decomposed by the supplied myocardial layer, the transmurally varying influence of the contraction of coronary flow is clearly seen (Fig. 3d). Opposite trends are observed during systole in the subendocardial (reversed) and subepicardial (augmented) flows, while the mid-myocardial layer features a relatively constant anterograde flow. Since the total inflow is always positive, this trend can be interpreted as a cardiac phase-dependent redistribution of flow between the layers. This mechanism has been reported by experimental studies (Rovai et al. 1989) and is also reflected by the segment-wise flow and fluid mass variation as shown in Fig. 3e, f. The time lag of fluid mass behind flow is another manifestation of the vascular compliance, showing for example that forward subendocardial flow which begins during early diastole requires until late diastole to replenish the fluid deficit inherited from the previous systole.

Further qualitative agreements are found with regards to experimental observations, namely that $p_\mathrm{pore}$ may exceed $p_{lv}$ locally (Westerhof 1990), $p_\mathrm{coro}$ features a dicrotic notch, and the reversal of cardiac outflow $Q_{ao}$ following aortic valve closure. On the other hand, the difference between $p_{lv}$ and $p_\mathrm{coro}$ is less than the range observed experimentally, leading to an absence of a crossover point of the two pressures at late systole. This indicates an insignificant pressure gradient across the aortic valve (and into coronary ostium) resulting from suboptimal valve model characterisation. However, the cWIA results are expected to be minimally affected by these since the time course of *dP* remains largely unchanged.

In the current analysis, the proportional cumulative areas of the individual waves (‘% wave area’) are used as the principal index for comparison with experimental data due to its independence on the cardiac load. Furthermore, our previous work has shown that peak magnitude of the wave is highly sensitive to the standard post-processing procedure, and thus is unsuitable for robust comparison (Rivolo et al. 2014).

The baseline WIA (Fig. 4; Table  2) shows overall an accurate model reproduction of the measured % area of individual waves. The two largest waves (⑤ backward suction wave & ② forward pushing wave) occupy a greater proportion than the mean values reported in Davies et al. (2006); however, given that their ratio is almost identical to the reported value (${\approx }1.35$), the difference may be attributed to the % errors in the other, less significant waves. The largest difference was found in the ③ late backward pushing wave, which is a reflection of the dominant forward pushing wave and thus is the wave which is most sensitive to the internal wave transmission characteristics of the vascular network. The underestimation of this wave is expected to have artificially inflated the % of all other waves. Often in the literature, it has been found that the early and late backward pushing waves (① and ③) cannot be reliably distinguished, and thus only the combined value was reported (Davies et al. 2011; Rolandi et al. 2012; Silva et al. 2013). With this approach, the model currently underestimates the observation by about $14.5\,\%$ ($22.4\,\%$ vs $7.9\,\%$). The ⑥ late forward pushing wave was overestimated, but expectedly due to the fully reflecting boundary condition imposed at the coronary ostium.

**Fig. 6:**
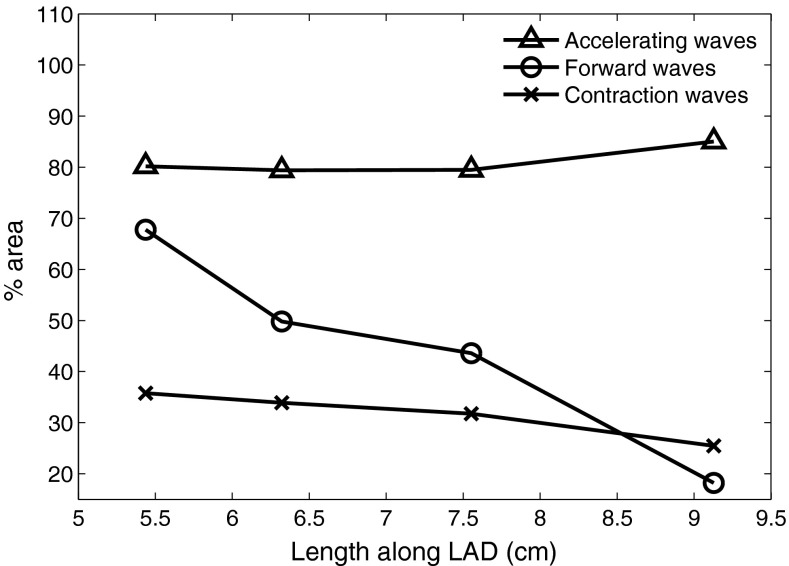
Changes in % area indices along distal LAD

The % wave areas can be grouped and summed according to contraction (①, ②, ③) versus relaxation phase, accelerating (②, ⑤, ⑥) versus decelerating, or forward (②, ④, ⑥) versus backward-travelling tendencies. As with the individual % areas these measures exhibit a dependence on the sampling location along the vessel. Figure 6 shows the variation of these indices along the distal half of the LAD where, notably, the total forward-travelling % wave area can be seen to diminish with distance. Conversely this implies that the total % wave energy balance is shifting from the forward to the backward waves distally towards the microcirculation.

**Fig. 7:**
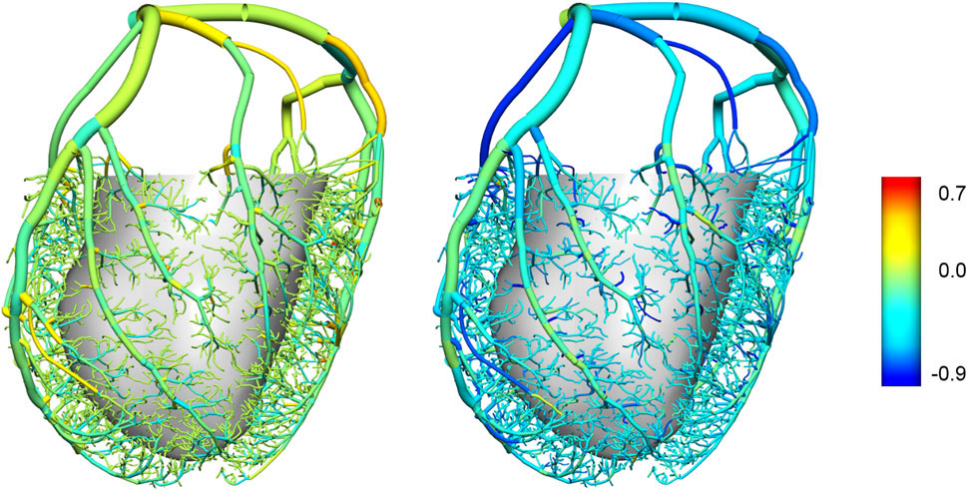
Forward (*left*) and backward (*right*) linear reflection coefficients under a constant wave speed of $15\,\text {m}\,\text {s}^{-1}$

These results must, however, be interpreted with caution and experimentally verified, due to the lack of specificity in network parameter tuning in the current model. The vascular mesh employed in this work lacks tapering as explained, which would result in a lack of wave reflections. In addition, wave speed governs the characteristic impedance of each vessel and, under a network setting a mismatch of impedances at a junction will lead to unphysiological wave reflections. Using a linearised analysis, the reflection coefficient of a pressure perturbation can be defined as (Sherwin et al. 2003)

65 $$\begin{aligned} R_f = \frac{{}^{A_o^1}\!/_{c_o^1}-{}^{A_o^2}\!/_{c_o^2}-{}^{A_o^3}\!/_{c_o^3}}{{}^{A_o^1}\!/_{c_o^1}+{}^{A_o^2}\!/_{c_o^2}+{}^{A_o^3}\!/_{c_o^3}} \end{aligned}$$

where the superscript refers to the parent (1) or daughter segments (2,3). While it has long been suggested that coronary junctions are well matched $({\text {i.e.}}\, R_f=0)$ for forward-travelling waves ( Arts et al 1979), the more recently identified prominence of the backward-travelling waves and the resultant trade-off between forward and backward matching have not been reconciled experimentally into a unified description. The forward and backward reflection coefficients under the constant wave speed regime are illustrated in Fig. 7. Coincidentally, the forward reflection coefficients are predominantly near 0 under this simple assumption while the backward-travelling waves mostly encounter negative coefficients due to the expanding area ratio. Our preliminary investigations indicate that when the wave speed is allowed to vary segment-to-segment, a reduction in the backward reflectances while simultaneously preserving the forward reflectance requires a significant progressive increase in the distal wave speed, unless one of the daughter vessels is of much larger calibre than the other.

### Wave intensity analysis

#### Modulators of coronary waves

*Contraction parameters* The wave intensity profiles were sensitive to both the shape of the tension transient as well as the QRS duration (QRSd). However, the dependence on QRSd was only an indirect one through modulation of the aortic sinus pressure. The reduction of QRSd to $0\,\text {m}\,\text {s}$ did not cause a significant difference in the shape of the contraction waves (①-③), neither was the timing of these waves altered as the timing of ejection was preserved under new activation sequence (Fig. 5, first column), whereas the accelerated rate of relaxation led to an augmented backward suction wave. On the other hand, a faster contraction and slower relaxation led to a change in all six waves (second column), correspondingly increasing the % area of contraction waves and reducing relaxation waves. An expanded analysis is described in Sect. 4.2.2.

*Haemodynamic parameters* A consequence of modifying $\alpha$ in the current model is the modulation of the friction coefficient through the factor $\frac{\alpha }{\alpha -1}$ [see Eq. (48)]. The result of perturbation to $\alpha =1.1$ from 1.05 was an approximate halving of the friction coefficient (third column), which increased the magnitude of all waves. This change was caused mostly through the increased flow rate in the vessels throughout the whole cycle, but affected the relaxation waves most significantly since the vascular resistance was at its minimum and the most rapid inflow gradient could be produced then. The effect of increasing the venous outflow pressure was much less pronounced (fourth column), with a near-uniform downward and upward shift in coronary velocity and pressure respectively but no change in the wave magnitudes. This is due to the fact that wave intensity is solely a function of the rates of change in velocity and pressure and has no dependence on their magnitude. Modifying the wave speed of the vessels to $10\,\text {m}\,\text {s}^{-1}$ resulted in an insignificant change to the wave intensity as a uniform scaling of wave speed preserves reflection coefficients as can be seen in (65). Also, due to the comparatively short vascular path lengths even a change of $-5\,\text {m}\,\text {s}^{-1}$ in the wave speed translated to only a few extra milliseconds in the wave propagation times.

**Fig. 8:**
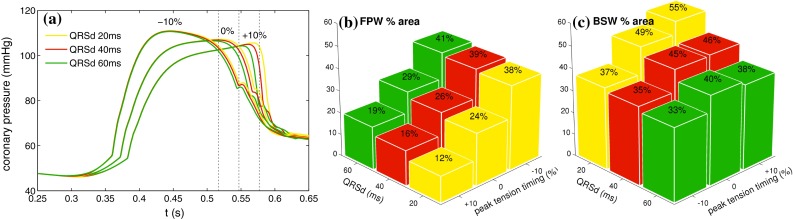
Wave sensitivity to contraction parameters **a** coronary pressure sampled at 6 cm along LAD. Modifying the time course of active tension resulted in a much greater effect on the pressure trace compared to changes in QRSd. Shown in *dotted lines* are the timing of the maximal tension, for a myocyte which is activated at $t=0.3$ s **b** forward pushing wave (FPW) % area shows a relatively linear dependence on both peak tension time and activation synchrony **c** backward suction wave (BSW) % area is positively correlated with peak tension time under synchronous activation, but the maximal rise associated with the rapid relaxation rate is lost with increasing level of dyssynchrony

*Aortic valve parameters* The modification of the aortic valve flow dynamics in Eq. (56) was a necessary element in tuning the forward wave intensities. Clearly, capturing the dynamics of the aortic sinus/valve region with a zero-dimensional model imposes limitations—however, the modification does, to an extent, alleviate an otherwise serious flaw in the model. The results of the fifth column emulate the wave dynamics in the absence of this modification, through reducing the time constant $T_{ao}$ by a factor of 10. The abrupt closure of the aortic valve leads to brief, but much steeper rates of rise in both coronary pressure and velocity, leading to around a fortyfold increase in the peak of the late forward pushing wave. While the changes to other waves are much less pronounced, their % areas collectively reduce due to the greatly expanded proportion of the forward pushing wave.

#### Contraction, relaxation and coronary waves

The results of the parameter sensitivity analysis indicate that coronary waves are strongly dependent on the relative rates of activation and relaxation, the synchrony of contraction and the time course of aortic sinus pressure. The codependency of these factors is further examined in Fig. 8 through simultaneous variation of the tension transient and QRS duration. The active tension parameters were adjusted to shift the timing of maximal tension by $\pm 10\,\%$ of the twitch duration, while keeping its magnitude constant. Figure 8a shows the changes in coronary pressure at the WI sampling location, where it can be seen that the time course of pressure is disproportionately sensitive to an advancement of peak tension in time, as opposed to when it is delayed. The effect of QRSd was more moderate, affecting most significantly the onset of the diastolic reduction in coronary pressure. These changes were directly reflected by the forward pushing wave % area (Fig. 8b), which exhibited a monotonic dependence on the tension transient (but with a greater sensitivity at $-10\,\%$ shift). It may appear counterintuitive that forward pushing wave decreases with improved synchrony—in terms of the actual magnitude, there was only a slight dependence on QRSd (possibly owing to the fact that activation gradient was imposed only transmurally in this model), and the observed trend was mostly a result of the expanded proportion of the waves that occur during relaxation.

In contrast, the dependence of backward suction wave % area on QRSd (Fig. 8c) was accompanied by changes in the wave magnitude and, under all variations of the tension transient, exhibited a reduction with increasing dyssynchrony. Furthermore, increasing level of dyssynchrony was found to be capable of overcoming even the accelerated relaxation rate of individual myocytes thereby reducing the % area of the backward suction wave as shown in Fig. 8c, top right row. Such a trend was not seen in the forward pushing wave, and highlights the stronger dependence of the backward waves on the intramyocardial stresses.

Increase in ⑤ backward suction wave has been reported to be associated with optimal biventricular pacing and the associated coronary flow increase in patients with dyssynchronous heart failure (Kyriacou et al. 2012). But the link between myocardial perfusion, coronary flow, and dyssynchronous heart failure is poorly understood at present, and the study of Kyriacou et al. (2012) remains the sole application of cWIA to heart failure to date. The key challenge is presented by the difficulty in separating the pathophysiological response from normal physiological response that arises as a result of altered electromechanics (Claridge et al. 2015). In this regard, the proposed model offers a new method by which to address these unanswered questions. They will be pursued in future investigations.

### Modulators of perfusion

Although WI was found to depend only weakly on the distal pressure $p_\mathrm{drain}$, both perfusion and fluid accumulation in the porous domain were significantly affected. An interesting observation, when $p_\mathrm{drain}$ was raised as shown in Fig. 5 (fourth column), was that the backward suction wave remained unmodified even when the pores were in a state of dilation at its onset. Under the current model assumptions, this implies a dominant role of the myocardial relaxation rate in producing the early diastolic suction over factors pertaining to the intracoronary haemodynamics. That is, wave behaviour was largely independent of the venous load.

The baseline mean perfusion rate of $6.2\,\text {mL}\,\text {g}^{-1}\,\text {min}^{-1}$ observed is significantly higher than the 1–2$\,\text {mL}\,\text {g}^{-1}\,\text {min}^{-1}$ range typically found in resting porcine experimental conditions (Fedor et al. 1978). However, the baseline parameterisation of $p_\mathrm{drain}$ was tuned to experimentally observed venous pressures, rather than to compensate for the lumped nature of the distal circulation in the current model. Indeed, when $p_\mathrm{drain}$ was raised to $37.5\,\text {mmHg} \,(5\,\text {kPa})$, more in line with the experimentally observed zero flow pressure (Bellamy 1978), the mean perfusion was reduced to $3.8\,mL\,g^{-1}min^{-1}$. In addition, the pressure drop along LAD was brought closer to a physiological level $(26\, {\text {to}}\, 15\,\text {mmHg}, {\text {from root to mid}})$. Likewise, in the absence of anatomical details, the baseline coupling interface resistances ($R_\mathrm{term}$ in (54)) were prescribed with a simple objective of minimising the uncontrolled wave reflections. When the observation that approximately half of the total pressure drop occurs between the arteriolar and capillary compartments under normal vasomotor tone (Chilian et al. 1989) is approximated by a tenfold increase in $R_\mathrm{term}$ along with raised $p_\mathrm{drain}$, physiological mean perfusion $(2.2\,\text {mL}\,\text {g}^{-1}\,\text {min}^{-1})$ and pressure drop along LAD are recovered $(7.9\,\text {mmHg})$. These changes occurred largely free of deviations from the baseline WI, except for a doubling of the ③ late backward pushing wave % area (see Table 2).

**Fig. 9:**
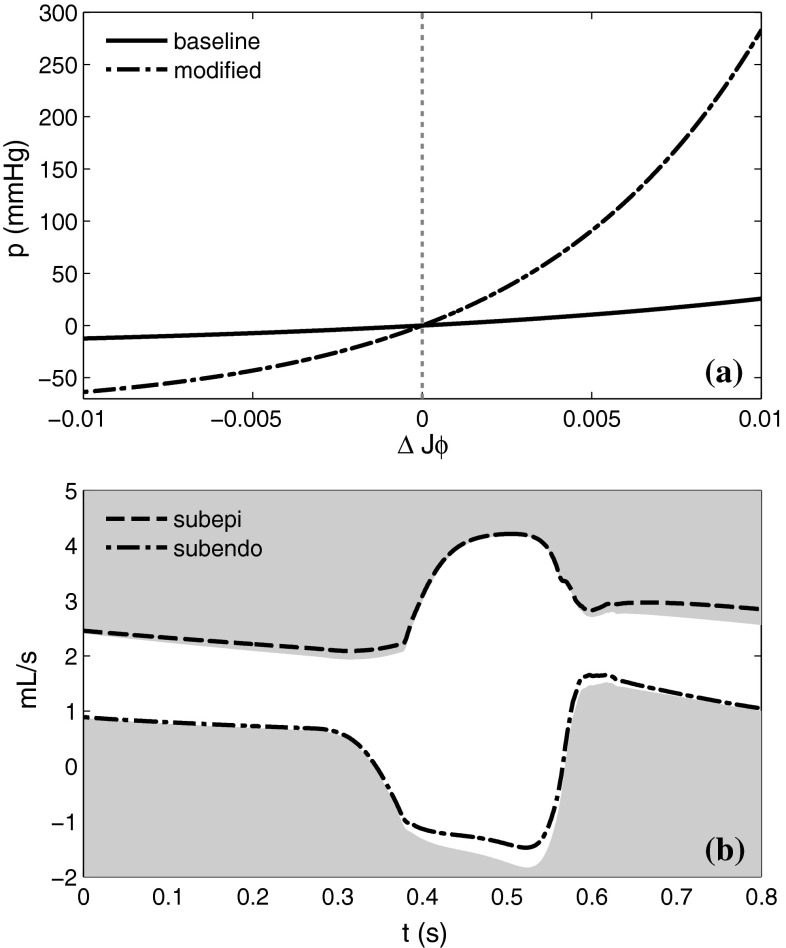
Effects of porous medium parameters **a** modified pressure–volume relation, **b** The modifications lead to an increase in systolic subendocardial and diastolic subepicardial flows. Shown in *grey* are baseline results for comparison

The consequence of modifying the porous compartment parameters $(q_1,q_2,q_3)$ was contrary to our expectation. The pressure–volume relationship of the porous domain was adjusted to reflect the arteriolar, rather than capillary properties as shown in Fig. 9a. As can be seen in 9b, its major effect was to raise the systolic subendocardial flow and diastolic subepicardial flow, leading to a sustained *increase* in the total coronary flow throughout the cardiac cycle. This was caused by the greater negative pressure generation in the compressed pores, thereby explaining the observed spatiotemporal variation in perfusion.

Where accurate reproduction of regional perfusion is concerned, these observations present a case for employing a multi-compartment porous domain framework (Michler et al. 2012; Hyde et al. 2014), which can distinguish the pressure and flow across the multiscale hierarchy of distal vessels. With a single-compartment model, reproduction of wave dynamics may require a compromise in the distal characteristics. However, we maintain that for WIA applications, the single-compartment model can sufficiently reproduce a broad range of behaviours. This is because waves which originate in the deep distal vessels have a limited influence over the waveforms observed in the large arteries due to wave-trapping and reflection effects (Davies et al. 2012).

### Age, wave speed and WIA

The present model-based investigations largely reaffirm the previously inferred wave-generating mechanisms in the experimental studies. In addition, further clarifying remarks can be made with respect to the age-related changes in wave characteristics. Even though age-related changes in coronary arteries may involve a myriad of mechanisms, wall stiffening had received a central emphasis. Although there is no reported evidence in the coronary arteries as yet, the increase in the pulse wave speed in the aorta with age is a well-documented phenomenon which may reasonably be expected to take place in the coronaries also. This being the case, Davies et al. (2006) found no correlation between the ⑤ backward suction wave and age, whereas a significant reduction in its % area was observed in patients with LV hypertrophy. Both of these observations are consistent with the simulation results presented here and reinforce the view that altered initiation of the suction wave by myocardial relaxation has a greater repercussion than its modifications during transmission to the proximal artery.

On the other hand, Davies et al. (2006) reported that ageing correlated positively with the ④ forward suction wave magnitude and offered two possible explanations. The ventricular–arterial hypothesis attributed the increased coronary stiffness and its ability to transmit a greater magnitude of energy into the vasculature. The aortic hypothesis ascribed the greater rates of rise, and correspondingly, the decline in the aortic pressure brought on by its own stiffening. The present work lends support to the dominant role of the second mechanism, especially given the absence of reported augmentation in the other forward-travelling waves.

### Shortcomings of the model

The present work is subjected to several shortcomings which should be addressed in future investigations. Of these, the design of a suitable constitutive law will require perhaps the most attention, as anisotropic poroelastic response in large strain regime is currently a sparsely explored area even in the field of biomedical engineering applications. Formal homogenisation approaches provide an alternative avenue, through which macroscopic description can be obtained from well-defined microscopic behaviours (see Davit et al. (2013) for an overview of methodologies). Even if these methods may lead to a computationally intensive coupled micro–macro problem, they may still play an important role in the validation of the practical approach adopted in this work.

The proposed model requires tuning of a large number of parameters to govern its behaviour. While global functional indices were shown to be physiological in this work, further experimental validation is key if the model is to be extended to predictive applications. Vascular network parameterisation presents additional difficulties in this regard due to their limited accessibility, and thus, theoretical developments are currently under way to complement these efforts. Balancing the distal resistance parameters (which modulate perfusion) with wave reflection behaviour on a vessel-wise basis was a challenging task, and should be explored further in future work.

In addition to the mechanisms described in Sect. 4.1, there are additional ones by which the coronary–myocardial coupling could take place that we have not explored in this work. Two prominent examples are (1) the direct extravascular compression on the explicitly represented vessels and (2) the effect of vessel motion on the flow and waveforms. However, we do not believe the omission of these mechanisms has resulted in significant errors for the following reasons. Of the 94,000 vessel segments reconstructed from the data, only ${<}4000$ proximal segments were retained for simulation.

Of these, those situated along the epicardium can be disregarded since it is known that systolic compression is insignificant there (Toyota et al. 2005). Considering the significant distal expansion in the number of vessels and total cross-sectional area (Kassab et al. 1993), the one or two generations of intramural vessels that are currently unaccounted for representing a tiny fraction of the total surface area available in the coronary network for vessel–myocardial interaction. Temporally, these transmurally extended vessels are $3\, {\text {to}}\, 5$ cm in length, such that waves initiating at the endocardium would experience only a $\sim 2$-ms delay, which is less than half the sampling interval at which most clinical WIA are conducted (Rivolo et al. 2014). Taken together, it is clear that in our model, the majority of the systolic compression occurs in, and is captured by, the region of vascular network represented by the poroelastic domain.

The impact of vessel motion is convoluted by numerous ways in which it may exert its influence on the vascular flow. Multiple simulation studies have revealed that the frequency and phase of the vessel motion relative to flow have the greatest impact, whereas the curvature variation of vessel segments was found to be of minimal consequence. The inertial effects were shown to be significant at 5 Hz vessel motion (Pivkin et al. 2005), whereas at 1 Hz they were negligible (Santamarina et al. 1998). The justification for applying motion at nearly five times the heart rate came from a frequency domain analysis of biplane-cineangiography images (Gross and Friedman 1998), although a later analysis of the data showed that around $85\,\%$ of the power was contained at or under 3 Hz (Moore et al. 2001), implying the modelling results are likely to have been exaggerated to a degree. In addition, the relative phase between vessel motion and flow was reported to be a major determinant of the resulting flow splitting at a bifurcation (Pivkin et al. 2005). The maximal effect was found when the flow was $270^\circ$ ahead of the outward ventricular motion, at which the flow ratio of the daughter vessels deviated around $\pm 25\,\%$ from the static myocardium / steady flow case. The deviation at $90\,\%$ phase difference was less than 5 %. We estimate the phase difference in our results to be in the range $0^{\circ } {-} 90^{\circ }$ since the majority of the anterograde arterial flow acceleration occurs during early diastole, to be followed by the bulk of the ventricular inflow. When compared to the ${\sim }10\,\%$ beat-to-beat variations in cathlab measurements and $\pm 20\,\%$ errors that may occur during WIA post-processing (Rivolo et al. 2014), we conclude that the existing evidence for cardiac-motion-induced effects in coronary flow indicates a secondary role that is within the order of magnitude of experimental error.

### Patient-specific modelling

While the translation of the present model framework to the clinics is beset by challenges in both data acquisition and modelling at current time, continuing advances in the medical imaging field present optimistic prospects. The recent introduction of 320-multidetector CT scanner combined with nonlinear iterative reconstruction now allows low-dose/high-resolution angiography for coronary vessel characterisation (Williams et al. 2013). Likewise, perfusion MR imaging is solidifying its gold standard status through phantom validation (Chiribiri et al. 2013) and patient trials (Greenwood et al. 2012), permitting high-resolution quantification of myocardial blood flow as recently demonstrated (Villa et al. 2015). Meanwhile, the scope of validation is currently being expanded through first-in-human simultaneous measurements of coronary pressure/velocity and ventricular pressure/volume by our group, which reveal a wealth of information regarding coronary–myocardial coupling. A reliable personalisation of a model is conditional upon the conformance between obtainable data and the complexity of the model. Although the eventual form of the model will likely entail a further reduction to the clinical essentials, it is likely to benefit from the closing gap between the data and model, as well as inverse parameter estimation techniques that have been successfully employed to myocardial mechanical characterisation (Asner et al. 2015).

## Conclusions

In this work, an *in silico* platform for coronary wave intensity analysis was developed and used to investigate the wave dependence on individual parameters underlying contraction, perfusion and systemic haemodynamic processes. To the authors’ knowledge, this is the first and only instance of such a study based on an integrated biophysical model of cardiac perfusion has been reported. While experimental coronary WIA is often afflicted by temporal waveform misalignment and motion-related issues which degrade the data quality, the present work offers a noise-free, repeatable and quantitative alternative. In addition, it overcomes the technological limitations in the bandwidth of measurements that can be simultaneously acquired, opening up new avenues in diagnostic index discovery and predictive clinical applications.

The simulation studies of wave mechanisms highlighted the direct and indirect relationships between variables underlying ventricular–aortic–coronary coupling. These findings will aid in the interpretation of clinical studies with respect to mechanisms of pathophysiology. Several milestones must be achieved prior to exploiting the full potential of the present work, which is the diagnostic application to patient data. Fine-tuning of vascular network parameters and validation against experimental data represent two immediate challenges that lie ahead. Efforts to address these issues are currently under way, through controlled *in vivo* experiments which build on the work of Schuster et al. (2010).

## Footnotes

1 In other studies, however, the deformation gradient of every individual constituent has been used in the kinematic description (e.g. see Boer (2005) and references therein), as the physical significance remains independent of this choice.

## References

[CR1] Aguado-Sierra J, Parker KH, Davies JE, Francis D, Hughes AD, Mayer J (2006) Arterial pulse wave velocity in coronary arteries. In: Engineering in medicine and biology society, 2006. EMBS’06. 28th annual international conference of the IEEE, IEEE, pp 867–870 10.1109/IEMBS.2006.259375 17946867

[CR2] Algranati D, Kassab GS, Lanir Y (2010). Mechanisms of myocardium–coronary vessel interaction. Am J Physiol Heart Circ Physiol.

[CR3] Arts T, Kruger RT, Gerven WV, Lambregts JA, Reneman RS (1979) Propagation velocity and reflection of pressure waves in the canine coronary artery propagation velocity and reflection of pressure waves in the canine coronary artery. Am J Physiol 237(4):H469–H474 10.1152/ajpheart.1979.237.4.H469 495732

[CR4] Asner L, Hadjicharalambous M, Lee J, Nordsletten D (2015) Stacom challenge: simulating left ventricular mechanics in the canine heart. In: Camara O et al (eds) Statistical atlases and computational models of the heart-imaging and modelling challenges. Springer, Berlin, pp 123–134

[CR5] Bellamy RF (1978). Diastolic coronary artery pressure–flow relations in the dog. Circ Res.

[CR6] Biot M (1972). Theory of finite deformations of porous solids. Indiana Univ Math J.

[CR7] Biot MA (1941). General theory of three-dimensional consolidation. J Appl Phys.

[CR8] de Boer R (2005). Trends in continuum mechanics of porous media.

[CR9] Bonet J, Wood RD (2008). Nonlinear continuum mechanics for finite element analysis.

[CR10] Bowen R (1980). Incompressible porous media models by use of the theory of mixtures. Int J Eng Sci.

[CR11] Bruinsma P, Arts T, Dankelman J, Spaan JAE (1988). Model of the coronary circulation based on pressure dependence of coronary resistance and compliance. Basic Res Cardiol.

[CR12] de Buhan P, Chateau X, Dormieux L (1998). The constitutive equations of finite strain poroelasticity in the light of a micro-macro approach. Eur J Mech A Solids.

[CR13] Chapelle D, Moireau P (2014). General coupling of porous flows and hyperelastic formulationsfrom thermodynamics principles to energy balance and compatible time schemes. Eur J Mech B Fluids.

[CR14] Chapelle D, Gerbeau JF, Sainte-Marie J, Vignon-Clementel IE (2010). A poroelastic model valid in large strains with applications to perfusion in cardiac modeling. Comput Mech.

[CR15] Chapman SJ, Shipley RJ, Jawad R (2008). Multiscale modeling of fluid transport in tumors. Bull Math Biol.

[CR16] Chilian WM, Layne SM, Klausner EC, Eastham CL, Marcus ML (1989). Redistribution of coronary microvascular resistance produced by dipyridamole. Am J Physiol Heart Circ Physiol.

[CR17] Chiribiri A, Schuster A, Ishida M, Hautvast G, Zarinabad N, Morton G, Otton J, Plein S, Breeuwer M, Batchelor P (2013). Perfusion phantom: An efficient and reproducible method to simulate myocardial first-pass perfusion measurements with cardiovascular magnetic resonance. Magn Reson Med.

[CR18] Cioranescu D, Donato P (1999). An introduction to homogenization.

[CR19] Claridge S, Chen Z, Jackson T, Sammut E, Sohal M, Behar J, Razavi R, Niederer S, Rinaldi CA (2015). Current concepts relating coronary flow, myocardial perfusion and metabolism in left bundle branch block and cardiac resynchronisation therapy. Int J Cardiol.

[CR20] Cook S (2011) Cardiovascular intervention in Europe 2009/2010. Presented at EuroPCR 2011

[CR21] Cookson A, Lee J, Michler C, Chabiniok R, Hyde E, Nordsletten D, Sinclair M, Siebes M, Smith N (2012). A novel porous mechanical framework for modelling the interaction between coronary perfusion and myocardial mechanics. J Biomech.

[CR22] Coussy O (1989). Thermodynamics of saturated porous solids in finite deformation. Eur J Mech A Solids.

[CR23] Coussy O (1995). Mechanics of porous continua.

[CR24] Coussy O (2004). Poromechanics.

[CR25] Davies JE, Whinnett ZI, Francis DP, Manisty CH, Aguado-Sierra J, Willson K, Foale RA, Malik IS, Hughes AD, Parker KH (2006). Evidence of a dominant backward-propagating suction wave responsible for diastolic coronary filling in humans, attenuated in left ventricular hypertrophy. Circulation.

[CR26] Davies JE, Sen S, Broyd C, Hadjiloizou N, Baksi J, Francis DP, Foale RA, Parker KH, Hughes AD, Chukwuemeka A (2011). Arterial pulse wave dynamics after percutaneous aortic valve replacement fall in coronary diastolic suction with increasing heart rate as a basis for angina symptoms in aortic stenosis. Circulation.

[CR27] Davies JE, Alastruey J, Francis DP, Hadjiloizou N, Whinnett ZI, Manisty CH, Aguado-Sierra J, Willson K, Foale RA, Malik IS (2012). Attenuation of wave reflection by wave entrapment creates a horizon effect in the human aortanovelty and significance. Hypertension.

[CR28] Davit Y, Bell CG, Byrne HM, Chapman LA, Kimpton LS, Lang GE, Leonard KH, Oliver JM, Pearson NC, Shipley RJ (2013). Homogenization via formal multiscale asymptotics and volume averaging: how do the two techniques compare?. Adv Water Resour.

[CR29] De Silva K, Foster P, Guilcher A, Bandara A, Jogiya R, Lockie T, Chowiencyzk P, Nagel E, Marber M, Redwood S (2013). Coronary wave energy a novel predictor of functional recovery after myocardial infarction. Circ Cardiovasc Interv.

[CR30] Dormieux L, Stolz C (1992). Variational approach for poroelastic medium. C R Acad Sci Paris.

[CR31] Federico S, Grillo A (2012). Elasticity and permeability of porous fibre-reinforced materials under large deformations. Mech Mater.

[CR32] Fedor JM, McIntosh DM, Rembert JC, Greenfield J (1978). Coronary and transmural myocardial blood flow responses in awake domestic pigs. Am J Physiol Heart Circ Physiol.

[CR33] Goyal A, Lee J, Lamata P, van den Wijngaard J, van Horssen P, Spaan J, Siebes M, Grau V, Smith NP (2013). Model-based vasculature extraction from optical fluorescence cryomicrotome images. IEEE Trans Med Imaging.

[CR34] Greenwood JP, Maredia N, Younger JF, Brown JM, Nixon J, Everett CC, Bijsterveld P, Ridgway JP, Radjenovic A, Dickinson CJ (2012). Cardiovascular magnetic resonance and single-photon emission computed tomography for diagnosis of coronary heart disease (CE-MARC): a prospective trial. Lancet.

[CR35] Gross MF, Friedman MH (1998). Dynamics of coronary artery curvature obtained from biplane cineangiograms. J Biomech.

[CR36] Hadjicharalambous M, Lee J, Smith NP, Nordsletten DA (2014). A displacement-based finite element formulation for incompressible and nearly-incompressible cardiac mechanics. Comput Methods Appl Mech Eng.

[CR37] Heusch G (2008). Heart rate in the pathophysiology of coronary blood flow and myocardial ischaemia: benefit from selective bradycardic agents. Br J Pharmacol.

[CR38] Hittinger L, Mirsky I, Shen YT, Patric TA, Bishop SP, Vatner SF (1995). Hemodynamic mechanisms responsible for reduced subendocardial coronary reserve in dogs with severe left ventricular hypertrophy. Circulation.

[CR39] Holzapfel GA, Ogden RW (2009). Constitutive modelling of passive myocardium: a structurally based framework for material characterization. Philos Trans Ser A Math Phys Eng Sci.

[CR40] Hyde ER, Cookson AN, Lee J, Michler C, Goyal A, Sochi T, Chabiniok R, Sinclair M, Nordsletten DA, Spaan J (2014). Multi-scale parameterisation of a myocardial perfusion model using whole-organ arterial networks. Ann Biomed Eng.

[CR41] Kassab GS, Rider CA, Tang NJ, Fung YC (1993). Morphometry of pig coronary arterial trees. Am J Physiol Heart Circ Physiol.

[CR42] Kerckhoffs R, Bovendeerd P, Prinzen F, Smits K, Arts T (2003). Intra- and interventricular asynchrony of electromechanics in the ventricularly paced heart. J Eng Math.

[CR43] Korakianitis T, Shi Y (2006). A concentrated parameter model for the human cardiovascular system including heart valve dynamics and atrioventricular interaction. Med Eng Phys.

[CR44] Koudstaal S, Lorkeers J, Sanne J, Slochteren FJ, Spoel TI, Hoef TP, Sluijter JP, Siebes M, Doevendans PA, Piek JJ (2013). Assessment of coronary microvascular resistance in the chronic infarcted pig heart. J Cell Mol Med.

[CR45] Kyriacou A, Whinnett ZI, Sen S, Pabari PA, Wright I, Cornelussen R, Lefroy D, Davies DW, Peters NS, Kanagaratnam P (2012). Improvement in coronary blood flow velocity with acute biventricular pacing is predominantly due to an increase in a diastolic backward-travelling decompression (suction) waveclinical perspective. Circulation.

[CR46] Lee J, Smith N (2008). Development and application of a one-dimensional blood flow model for microvascular networks. Proc Inst Mech Eng H J Eng Med.

[CR47] Lee J, Smith NP (2012). The multi-scale modelling of coronary blood flow. Ann Biomed Eng.

[CR48] Lee J, Cookson A, Roy I, Kerfoot E, Asner L, Vigueras G, Sochi T, Michler C, Smith N, Nordsletten D (2016) Multi-physics computational modeling in CHeart. SIAM J Sci Comput (accepted)

[CR49] Maron MS, Olivotto I, Maron BJ, Prasad SK, Cecchi F, Udelson JE, Camici PG (2009). The case for myocardial ischemia in hypertrophic cardiomyopathy. J Am Coll Cardiol.

[CR50] Michler C, Cookson A, Chabiniok R, Hyde E, Lee J, Sinclair M, Sochi T, Goyal A, Vigueras G, Nordsletten D (2012). A computationally efficient framework for the simulation of cardiac perfusion using a multi-compartment darcy porous-media flow model. Int J Numer Methods Biomed Eng.

[CR51] Moore JE, Weydahl ES, Santamarina A (2001). Frequency dependence of dynamic curvature effects on flow through coronary arteries. J Biomech Eng.

[CR52] Mynard JP, Penny DJ, Smolich JJ (2014). Scalability and in vivo validation of a multiscale numerical model of the left coronary circulation. Am J Physiol Heart Circ Physiol.

[CR53] Nakanishi K, Fukuda S, Shimada K, Miyazaki C, Otsuka K, Maeda K, Miyahana R, Kawarabayashi T, Watanabe H, Yoshikawa J, Yoshiyama M (2012). Impaired coronary flow reserve as a marker of microvascular dysfunction to predict long-term cardiovascular outcomes, acute coronary syndrome and the development of heart failure. Circ J.

[CR54] Nesto RW, Kowalchuk GJ (1987). The ischemic cascade: temporal sequence of hemodynamic, electrocardiographic and symptomatic expressions of ischemia. Am J Cardiol.

[CR55] Parker KH (2009). An introduction to wave intensity analysis. Med Biol Comput.

[CR56] Parker KH, Jones CJH (1990). Forward and backward running waves in the arteries. Analysis using method of characteristics. J Biomech Eng.

[CR57] Pivkin I, Richardson P, Laidlaw D, Karniadakis G (2005). Combined effects of pulsatile flow and dynamic curvature on wall shear stress in a coronary artery bifurcation model. J Biomech.

[CR58] Rajappan K, Rimoldi OE, Camici PG, Bellenger NG, Pennell DJ, Sheridan DJ (2003). Functional changes in coronary microcirculation after valve replacement in patients with aortic stenosis. Circulation.

[CR59] Rivolo S, Asrress KN, Chiribiri A, Sammut E, Wesolowski R, Bloch L, Grondal A, Honge J, Kim W, Marber M, Redwood S, Nagel E, Smith NP, Lee J (2014). Enhancing coronary wave intensity analysis robustness by high order central finite differences. Artery Res.

[CR60] Rohan E (2006). Modeling large-deformation-induced microflow in soft biological tissues. Theor Comput Fluid Dyn.

[CR61] Rohan E, Cimrman R (2010). Two-scale modeling of tissue perfusion problem using homogenization of dual porous media. Int J Multiscale Comput Eng.

[CR62] Rohan E, Lukes V (2011) Computational homogenization for two-scale modeling of large deforming perfused tissues. In: Onate E, Owen DRJ (eds) XI international conference on computational plasticity. Fundamentals and applications, Lc

[CR63] Rolandi MC, Nolte F, van de Hoef TP, Remmelink M, Baan J, Piek JJ, Spaan JaE, Siebes M (2012). Coronary wave intensity during the Valsalva manoeuvre in humans reflects altered intramural vessel compression responsible for extravascular resistance. J Physiol.

[CR64] Rossi GE (2007) Numerical simulation of perfusion in the beating heart. Master’s thesis, Politecnico di Milano

[CR65] Rovai D, L’Abbate A, Lombardi M, Nissen SE, Marzilli M, Distante A, Ferdeghini EM, DeMaria AN (1989). Nonuniformity of the transmural distribution of coronary blood flow during the cardiac cycle. In vivo documentation by contrast echocardiography. Circulation.

[CR66] Santamarina A, Weydahl E, Siegel JM, Moore JE (1998). Computational analysis of flow in a curved tube model of the coronary arteries: effects of time-varying curvature. Ann Biomed Eng.

[CR67] Schuster A, Grünwald I, Chiribiri A, Southworth R, Ishida M, Hay G, Neumann N, Morton G, Perera D, Schaeffter T, Nagel E (2010). An isolated perfused pig heart model for the development, validation and translation of novel cardiovascular magnetic resonance techniques. J Cardiovasc Magn Reson.

[CR68] Sherwin SJ, Franke V, Peiró J, Parker K (2003). One-dimensional modelling of a vascular network in space-time. J Eng Math.

[CR69] Shipley RJ, Chapman SJ (2010). Multiscale modelling of fluid and drug transport in vascular tumours. Bull Math Biol.

[CR70] Siebes M, Kolyva C, Verhoeff BJ, Piek JJ, Spaan JA (2009). Potential and limitations of wave intensity analysis in coronary arteries. Med Biol Eng Comput.

[CR71] Smith AF, Shipley RJ, Lee J, Sands GB, Legrice IJ, Smith NP (2014) A model-based quantification of network permeability in the rat coronary microcirculation. Ann Biomed Eng (in review)

[CR72] Smith N, Pullan AJ, Hunter PJ (2002). An anatomically based model of transient coronary blood flow in the heart. SIAM J Appl Math.

[CR73] Spaan J (1985). Coronary diastolic pressure–flow relation and zero flow pressure explained on the basis of intramyocardial compliance. Circ Res.

[CR74] Spaan JAE, ter Wee R, van Teeffelen JWGE, Streekstra G, Siebes M, Kolyva C, Vink H, Fokkema DS, VanBavel E (2005). Visualisation of intramural coronary vasculature by an imaging cryomicrotome suggests compartmentalisation of myocardial perfusion areas. Med Biol Eng Comput.

[CR75] Toyota E, Ogasawara Y, Hiramatsu O, Tachibana H, Kajiya F, Yamamori S, Chilian WM (2005). Dynamics of flow velocities in endocardial and epicardial coronary arterioles. Am J Physiol Heart Circ Physiol.

[CR76] Truesdell C (1957). Sulle Basi Della Termomeccanica. Rend Accad Lincei.

[CR77] Truesdell C, Noll W (1965). The non-linear field theories of mechanics.

[CR78] Van Kerckhoven R, van Veghel R, Saxena PR, Schoemaker RG (2004). Pharmacological therapy can increase capillary density in post-infarction remodeled rat hearts. Cardiovasc Res.

[CR79] Villa AD, Sammut E, Zarinabad N, Carr-White G, Lee J, Bettencourt N, Razavi R, Nagel E, Chiribiri A (2015). Microvascular ischemia in hypertrophic cardiomyopathy: new insights from high-resolution combined quantification of perfusion and late gadolinium enhancement. J Cardiovasc Magn Reson.

[CR80] van de Vosse FN, Stergiopulos N (2011). Pulse wave propagation in the arterial tree. Annu Rev Fluid Mech.

[CR81] Westerhof N (1990). Physiological hypotheses-intramyocardial pressure. A new concept, suggestions for measurement. Basic Res Cardiol.

[CR82] Westerhof N, Boer C, Lamberts RR, Sipkema P (2006) Cross-talk between cardiac muscle and coronary vasculature. Physiol Rev 1263–1308. doi:10.1152/physrev.00029.2005 10.1152/physrev.00029.2005 17015490

[CR83] Westerhof N, Lankhaar JW, Westerhof BE (2009). The arterial Windkessel. Med Biol Eng Comput.

[CR84] Whitaker S (1986). Flow in porous media I: a theoretical derivation of Darcy’s law. Transp Porous Media.

[CR85] Williams MC, Cruden NL, Uren NG, Newby DE (2013). A low-dose comprehensive cardiac ct protocol assessing anatomy, function, perfusion, and viability. J Cardiovasc Comput Tomogr.

[CR86] Zhang L, Allen J, Hu L, Caruthers SD, Wickline SA, Chen J (2013). Cardiomyocyte architectural plasticity in fetal, neonatal, and adult pig hearts delineated with diffusion tensor MRI. Am J Physiol Heart Circ Physiol.

